# Alternative splicing regulates stochastic NLRP3 activity

**DOI:** 10.1038/s41467-019-11076-1

**Published:** 2019-07-19

**Authors:** Florian Hoss, James L. Mueller, Francisca Rojas Ringeling, Juan F. Rodriguez-Alcazar, Rebecca Brinkschulte, Gerald Seifert, Rainer Stahl, Lori Broderick, Chris D. Putnam, Richard D. Kolodner, Stefan Canzar, Matthias Geyer, Hal M. Hoffman, Eicke Latz

**Affiliations:** 1 Institute of Innate Immunity, University Hospital, University of Bonn, 53127 Bonn, Germany; 2 University of California, San Diego School of Medicine, La Jolla, CA 92093 USA; 3 Gene Center, Ludwig-Maximilians-Universität München, 81377 Munich, Germany; 4 Institute of Structural Biology, University Hospital, University of Bonn, 53127 Bonn, Germany; 5 Medical Faculty, Institute of Cellular Neurosciences, University of Bonn, 53127 Bonn, Germany; 6 Ludwig Institute of Cancer Research, San Diego branch, La Jolla, CA 92093 USA; 7 Division of Infectious Diseases and Immunology, University of Massachusetts Medical School, Worcester, MA 01655 USA; 8 German Center for Neurodegenerative Diseases, 53127 Bonn, Germany

**Keywords:** RNA splicing, Inflammasome, Innate immunity, NOD-like receptors

## Abstract

Leucine-rich repeat (LRR) domains are evolutionarily conserved in proteins that function in development and immunity. Here we report strict exonic modularity of LRR domains of several human gene families, which is a precondition for alternative splicing (AS). We provide evidence for AS of LRR domain within several Nod-like receptors, most prominently the inflammasome sensor NLRP3. Human NLRP3, but not mouse NLRP3, is expressed as two major isoforms, the full-length variant and a variant lacking exon 5. Moreover, NLRP3 AS is stochastically regulated, with NLRP3 ∆ exon 5 lacking the interaction surface for NEK7 and hence loss of activity. Our data thus reveals unexpected regulatory roles of AS through differential utilization of LRRs modules in vertebrate innate immunity.

## Abstract (web summary)

Leucine-rich repeat (LRR) domains are commonly present in immune regulatory proteins. Here the authors show that LRR exonic modularity and alternative splicing of an LRR-containing protein, NLRP3, modulate the ratio of functional/afunctional NLRP3 isoforms to instill a stochastic regulation of NLRP3-mediated inflammation and innate immunity.

## Introduction

Innate immune cells rely on germ-line encoded signaling receptors, so called pattern-recognition receptors (PRRs), to sense tissue dyshomeostasis induced by infection or caused by metabolic defects and other injurious insults^1,2^. The interpretation of these physico-chemically diverse molecular cues by immune cells is influenced and shaped by numerous mechanisms including chemokine, cytokine, or G protein-coupled receptors^3–5^. Innate immune cells must integrate those signals and generate a tissue context adjusted inflammatory response aimed at the destruction of microbes and repair of damaged tissue, while preventing further damage caused by an inappropriately robust response.

NLR proteins have an especially wide spectrum of functions, as they can act as cytosolic sensors of microbial molecules^6^, support immune homeostasis in various tissues^7^, and play critical roles in reproduction and embryogenesis^8^. Upon activation, some members of the NLR family (e.g. NLRP3 and NLRC4) recruit multi-protein signaling platforms, so called inflammasomes. NLRP3 consists of three functional domains, the N-terminal pyrin domain (PYD), the NOD (also known as NACHT), and the C-terminal LRR domain^9^. Inflammasome formation can be induced by different receptors, however, they converge on the level of ASC which serves as a platform for caspase-1 activation^10^. Active caspase-1 cleaves pro-IL-1β and pro-IL-18 and induces the release of their mature forms, which exert potent pro-inflammatory effects^11^. Furthermore, the activation of caspase-1 results in an inflammatory type of cell death termed pyroptosis, which requires gasdermin-D (GSDMD) cleavage^12^. NLRP3 is triggered by asbestos, silica and other particular matter and is involved in gout, atherosclerosis, and several neurodegenerative diseases^13^. Due to its involvement in multiple diseases, NLRP3 has become one of the most studied PRRs. Nevertheless, the molecular mechanisms of NLRP3 activation and regulation remain only partially understood. A possible common upstream activation mechanism involves potassium efflux from the cell^14,15^. Yet, potassium-independent activators of the NLRP3 inflammasome were postulated as well^16,17^. Recently, the NIMA related Kinase 7 (NEK7) was found to interact with NLRP3 as a precondition of its activation^18–20^.

Another important, albeit less studied, mechanism of signaling regulation is governed by alternative pre-mRNA splicing (AS). During AS introns are removed and some protein coding exons can be excluded from the assembled mature mRNAs allowing for the generation of various mature mRNAs from the same DNA template. AS can dramatically increase transcriptome complexity and allows for the generation of various proteins from individual genes with altered or distinct functions^21^. The proportion of alternatively spliced genes has steadily increased during eukaryotic evolution suggesting that AS contributed to gain of organismal complexity during evolution. In humans, at least 95% of multi-exonic genes undergo alternative splicing^22^ and AS is particularly widespread in the nervous and immune systems^23,24^. There is increasing evidence that AS contributes both to the complexity of immune responses in various kingdoms^25^ and to the development of various diseases^26^. Alternatively spliced proteins can vary in their domain composition, sub-cellular localization or binding to ligands. Different isoforms can even act like unrelated proteins and are frequently characterized by significantly different interaction profiles^27^. In the adaptive immune system, different isoforms of CD45 and CD3ζ shape T-cell activity^24,28^. While traditionally associated with adaptive immunity, AS also provides mechanisms for PRR signaling. For instance, a short isoform of MD-2 acts as a negative feed-back regulator of TLR4^29^.

Similar to other evolutionarily conserved PRRs, NLRP3 contains structural elements that form a characteristic α/β horseshoe fold consisting of leucine-rich repeats (LRR) motifs. LRR domains are composed of repeating 20–30 amino acid stretches rich in leucine, and each LRR unit typically contains a beta strand-turn-alpha helix structure. LRR motifs confer the solenoid structure and LRR domains provide the binding sites for a broad spectrum of ligands, including proteins^30^, lipids^31^, nucleic acids^32^, and bases^33^. Notably, the adaptive immune system of jawless vertebrates lack the recombinatorial antigen receptors present in jawed vertebrates, yet instead relies on lymphocyte receptors composed of variable LRRs, which are assembled through somatic recombination. Variable lymphocyte receptors (VLRs) represent the adaptive arm of the immune system demonstrating the LRRs versatility in interacting with a broad spectrum of molecular entities^34^. In jawed vertebrates the LRR domain has a special role in the regulation of NLR function. For example, crystallization of the NLRC4 receptor showed that the LRR sequesters NLRC4 in an auto-inhibited state and interacts with protein ligands^35^. Similar mechanisms have been proposed for NLRP3 but have not been confirmed.

Here, we describe how AS of LRR genes drives the diversity of PRRs and we analyze in detail how different NLRP3 isoforms affect inflammasome activation. We identify that a prominent splice form of NLRP3 cannot interact with NEK7 and therefore is unable to form an inflammasome under conditions where the full-length NLRP3 is fully functional. We propose that AS may contribute to stochastic activatability of NLRP3 and suggest that AS of LRRs in NLRs regulates human innate immune responses.

## Results

### Multiexonic organization of the LRR domain in the NLR family

**Fig. 1:**
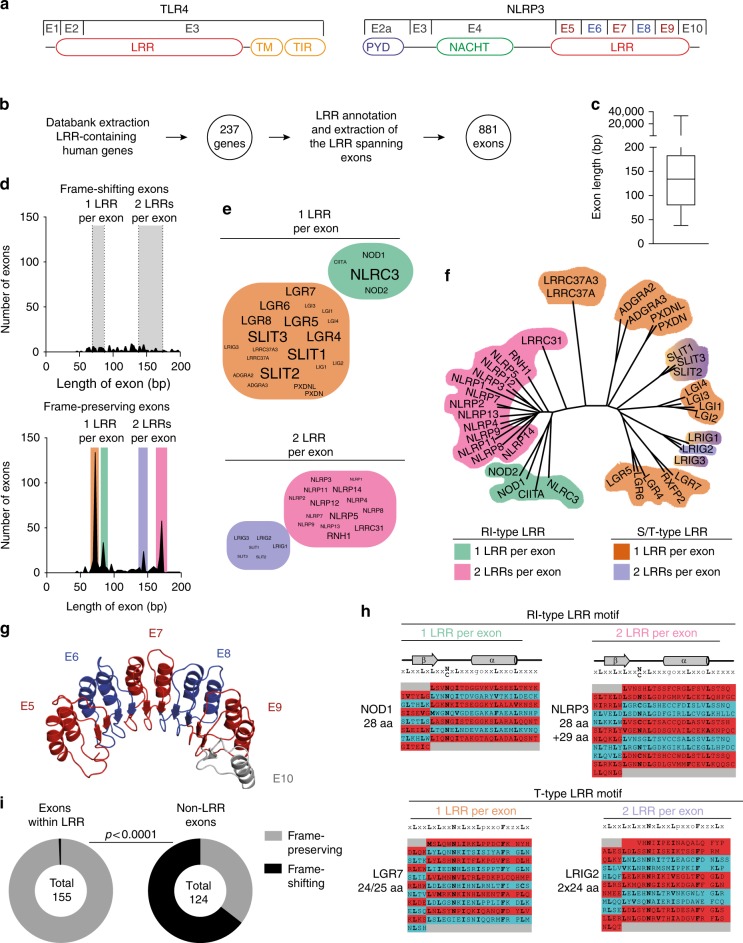
LRR domains of the NLR family have a conserved multi-exon organization. **a** Scheme of domain and exon distribution in TLR4 and NLRP3. **b** Workflow for the selection of LRR exons used in **c**–**f**. **c** Length distribution of exons extracted in **b**. Box indicates 25th to 75th percentile, the middle line indicates the median, whiskers indicate min and max values. **d** All exons up to 200 bp in length plotted for their frequency distribution. Exons were divided in frame-shifting or frame-preserving. The typical length of LRRs (23 to 29 aa) is indicated as gray boxes. Colored boxes in the lower panel match assignment of groups in the following subfigures. **e** Genes in word clouds represent those genes whose exons contribute to the respective peaks in **d**. Word size is linearly dependent to the number of contributing exons. **f** Phylogenetic analysis of all genes contributing to the 4 major peaks in **d**. **g** Model of the NLRP3 LRR based on the human ribonuclease inhibitor LRR crystal structure. **h** LRR consensus sequences and structural alignments for the four groups identified in **d**–**f**. **i** Quantification of all LLR exons vs. non-LRR exons of all NLRs which would be frame-shifting if alternatively spliced. Independence of distribution was calculated using two-tailed Fischer’s exact test. See also Supplementary Fig. 1. Source data are provided as a Source Data file

In TLRs, the majority of the LRR domain is encoded by a long exon, which also encodes other functional units such as the transmembrane and TIR domains. In contrast, the LRR domain in NLR family members is typically encoded by multiple consecutive short exons, reminiscent of the short LRR encoding gene segments found in the jawless vertebrate adaptive immune system (Fig. 1a). We hypothesized that the characteristic exonic organization found in the LRRs of the NLR family might be conserved in LRR encoding gene families and that differential splicing within the LRR could contribute to functional diversity of encoded proteins.

We performed a database analysis of all human genes annotated to encode LRR domains and analyzed the length distribution of the respective exons (Fig. 1b, c). Indeed, more than half of all LRR encoding exons ranged between 75 and 200 bp, which is the size range found in the LRR encoding exons of the NLR family. A few exons picked up by the analysis are terminal exons, which stretch far into the non-coding UTR of genes and give only seemingly rise to large proteins. Exon skipping by AS can lead to disruption of the original open reading frame, often resulting in truncated protein products or nonsense-mediated decay. We classified all detected exons smaller than 200 bp as either frame-shifting or frame-preserving, and plotted exon length versus their frequency. The vast majority was found to be frame-preserving, suggesting that AS would also preserve protein translation (Fig. 1d). Furthermore, exons with defined lengths that correspond to one or more LRRs of a typical length (69–87 bp or 23–29 aa) were highly enriched, suggesting that an exon-LRR structure relationship evolved (Fig. 1d). We identified which genes contributed to the four major peaks of frame-preserving exon length (Fig. 1e) and performed a phylogenetic analysis on them (Fig. 1f). The peaks corresponded to distinct gene families with conserved LRR exon structures, suggesting conservation of exon structures compatible with AS of LRR domains in several gene families.

The high ligand diversity of the LRR-recombination based jawless vertebrate adaptive immune system and the exonic organization of the NLR LRRs might suggest a convergent evolution of different classes of immune receptors. Therefore, we focused our further analysis on the NLR LRRs, performed structural alignments for all NLR LRRs and modeled the potential structure of the NLRP3 LRR based on the human ribonuclease inhibitor (RI) crystal structure (Fig. 1g). The NLRP3 LRR is highly canonical. Every exon is exactly 171 bp long and encodes two LRR, strictly alternating between 28 and 29 aa, resulting in homogenous building blocks with a defined surface curvature^36^. Strikingly, the exon–exon border is always positioned at the exact same position within the β-sheet, a characteristic that is conserved across most NLRs. Most of them could be clearly assigned in one of two classes only based on their exon structure. The NLRP genes contain exons encoding two alternating LRRs of 28 and 29 aa, while most NOD and NLRC genes contain exons that encode one LRR of 28 aa per exon (Fig. 1h, Supplementary Fig 1a). This repetitive exon architecture is a necessary precondition for the generation of functional isoforms by AS. This is because the removal of one or several exons will produce a shorter LRR domain likely without interfering with the overall protein fold, as the hydrophobic residues forming the core of the LRR scaffold are conserved. Moreover, the LRR exons of the NLR proteins are strictly frame-preserving, while the large majority of exons outside of the LRR region would lead to a frameshift if alternatively spliced, indicating an evolutionary pressure to maintain the spliceability of the LRRs (Fig. 1i). This exon-LRR relationship is not only conserved within the NLR family but also within the protein families containing the S/T (bacterial/typical) class of LRRs (Fig. 1d–f, h). Together, this analysis implies that differential splicing of LRR modules could create diverse protein functions or could regulate the activity of human NLR family members.

### The splicing landscape of human NLRs

**Fig. 2:**
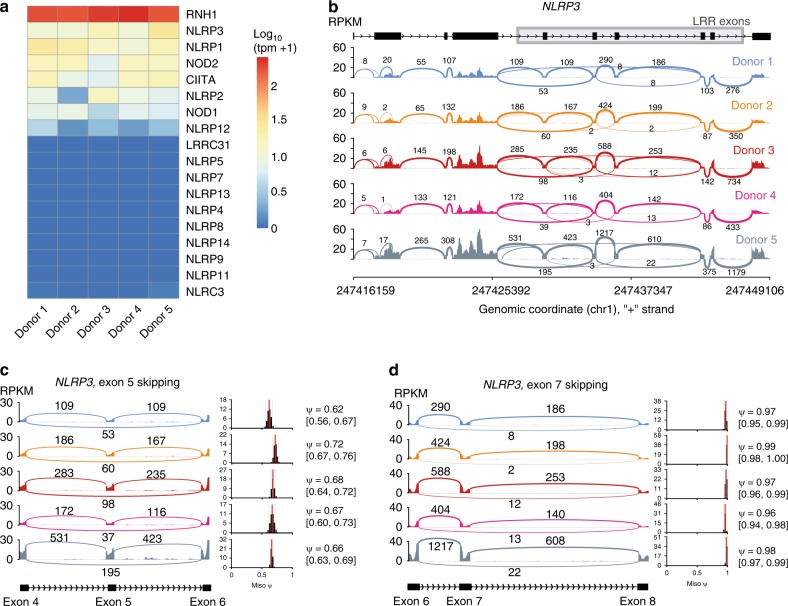
The splicing landscape of human NLRs. **a** Gene expression of all previously identified RI-type LRR encoding genes (Fig. 1d–f). **b** Sashimi plot of NLRP3 expressed in human monocyte-derived macrophages established from five healthy human blood donors created with MISO. Read frequency within exons is plotted as RPKM and exon spanning reads are labeled with the number of mapped reads. The NLRP3 gene structure is plotted above, with boxes indicating exonic regions and arrows within the intronic stretches indicating the reading directions. Short repetitive LRR exons are highlighted with a gray box. The genomic location is depicted below. **c**, **d** Sashimi plots as in **b**, focused on exons 4–5–6 and 6–7–8, respectively. MISO ψ (% spliced in) values (red bars in histogram) indicate the calculated frequency of exon inclusion. 95% confidence intervals are indicated as gray bars in the histogram. Ψ and CI values are listed as well numerically. See also Supplementary Fig. 2 and Supplementary Table 1. Source data are provided as a Source Data file

To analyze AS of human NLRs we performed RNAseq on human monocyte-derived macrophages (hMDMs) from five different donors. We aimed for a sequencing depth of 200 M paired-end reads per donor to allow for a reliable detection of alternative splicing events (for mapping summary see Supplementary Table 1). We concentrated on RI-type LRR genes, which are comprised mostly of NLRs. Prominent members of the NLR family are the inflammasome activating receptors, NLRP1, NLRP3, and NLRC4. However, NLRs are involved in diverse immune and non-immune processes and by far not all of them are expressed in macrophages. From all previously defined RI-type LRR genes (Fig. 1d–f), ribonuclease inhibitor (RNH1) was most highly expressed, followed by NLRP3, NLRP1, NOD2, CIITA, NLRP2, NOD1, and NLRP12 (Fig. 2a).

To monitor alternative splicing, we created sashimi plots for those NLRs expressed in macrophages. Sashimi plots show the read distribution along a gene locus and highlight exon-spanning reads as arcs (Fig. 2b and Supplementary Fig. 2a–g). To allow for reliable AS detection above splice noise, a high sequencing coverage is important. Therefore, we focused on NLRP3 as the most highly expressed NLR gene within this group.

Although AS events could be detected in all NLRs, the most prominent AS within the LRR region was observed in NLRP3 (Fig. 2b and Supplementary Fig 2). To further characterize NLRP3 splicing we calculated the exon inclusion levels (ψ = % spliced in) for exon 5 and 7 using MISO (Fig. 2c, d). The narrow confidence intervals of the ψ histograms indicate a high level of certainty for the inclusion frequency of the respective exon according to the read distribution. It is evident that exon 5 is consistently the most skipped exon (33%, 2.6% of exon 7), yet minor splicing differences could be observed between donors.

Although exon-spanning reads allow for a qualitative analysis of AS, a direct quantification of exon spanning reads would only be accurate for a perfectly uniform fragment distribution across a gene. However, this is not achievable as evidenced by the unequal read distribution within continuous long exons or intron-spanning reads of consecutive exons.

### The LRR domain of human *NLRP3* undergoes alternative splicing

**Fig. 3:**
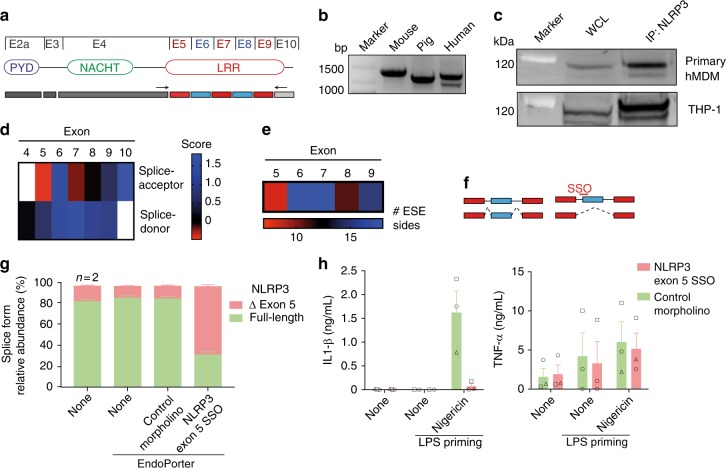
The LRR domain of human NLRP3 is subject to alternative splicing **a** Scheme of the NLRP3 exons and domains. Arrows indicate primers used in **b** and Supplementary Fig. 3a, b. **b** PCR of the NLRP3 LRR on cDNA isolated from LPS primed mouse BMDMs, pig and human PBMCs, respectively. Representative of at least three (mouse, human) or two (pig) individuals. **c** Immunoblot of human NLRP3 from primary human monocyte-derived macrophages (hMDM) or THP-1 cells. Either whole cell lysates or NLRP3 immunoprecipitates, using mAb targeted against the NACHT domain of NLRP3, were immunostained with a mAb targeted against the PYD to ensure NLRP3 specificity. Representative of two experiments. **d** Scores for the probability to function as splice acceptor and donor sites were calculated for all human NLRP3 LRR exon boundaries using SplicePort. **e** Number of exonic splice enhancer (ESE) sites within the exons of the LRR as predicted by RESCUE-ESE. **f** Scheme of a splice-switching oligo (SSO), blocking the spliceosomal access to an intron-exon boundary inducing AS. **g** Changes in the NLPR3 alternative splicing pattern of M0 hMDMs were induced with an exon 5 SSO. NLRP3 isoform expression analysis by qPCR. Mean and SEM of three donors (untreated: *n* = 2). **h** Cytokine secretion of morpholino-treated cells after priming with LPS (TNF) and nigericin-induced activation of the NLRP3 inflammasome (IL-1β). Mean and SEM of 3 donors (LPS only: *n* = 2). Each donor is plotted using a unique symbol shape. See also Supplementary Fig. 3. Source data are provided as a Source Data file

After initial characterization of the splicing patterns of the NLR gene family, we aimed to validate the RNAseq results and to decipher whether differential splicing can affect the protein function of NLRP3. We thus PCR amplified the LRR region of *NLRP3* transcripts by using primers flanking the LRR region (Fig. 3a). While the amplified product from human cells ran as double band on agarose gels, we were unable to detect any alternative splicing of the LRR region of *Nlrp3* in murine bone marrow-derived macrophages or pig PBMCs (Fig. 3b). The size difference of 171 bp between the two human amplicons corresponds to the size of a single LRR exon. We characterized the lower PCR band to encode for the greatest extend ∆ exon 5 isoform (Supplementary Fig. 3a).

To confirm the existence of a shorter splice variant of NLRP3 at the protein level, we next performed immunoprecipitation – Western blot analysis on primary hMDMs and THP-1 cells (Fig. 3c). To ensure that nonspecific Western blot bands are not mistaken for an NLRP3 isoform, we performed immunoprecipitations using an NLRP3 mAb targeted against the NACHT domain and used a different mAb targeted against the NLRP3 PYD to visualize NLRP3. Of note, NLRP3 splicing in mouse BMDMs was not detected at the RNA or protein level (Supplementary Fig. 3b, c), and we thus conclude that AS of NLRP3 is primarily found in human immune cells.

The described structural features of the NLRP3 LRR would allow every LRR exon to be spliced out equally well. However, our RNAseq and PCR analysis revealed a strong tendency for AS of exon 5. Certain sequence elements can be associated with the strength of a splice acceptor or donor site^37^. We used the SplicePort algorithm tool to analyze the strength of splice donor and acceptor sites in human NLRP3 pre-mRNA and identified exon 5 acceptor followed by exon 7 acceptor as the least likely to be used (Fig. 3d). Consistently, within the LRR of human NLRP3, the fewest exonic splice enhancer sites (ESEs) were detected in exon 5 using ESEfinder^38^ (Fig. 3e).

To analyze the prevalence of different isoforms more accurately, we performed qPCR analysis using specific primers for a given splice variant to minimize PCR bias of a long range PCR. Besides the full-length variant and ∆ exon 5, we probed as well for ∆ exon 7 and ∆ exon 5/7 (Supplementary Fig. 3d), as the most likely alternative splice events according to the SplicePort scores (Fig. 3d) and our RNAseq data (Fig. 2). As expected, the NLRP3 full-length variant was the major isoform and the splice variant lacking exon 5 was the second most abundant, while the isoforms lacking exon 7 and exon 5 and 7 were expressed one and two magnitudes of order lower regardless of the polarization state (M0, M1, and M2) of hMDMs (Supplementary Fig. 3e).

### NLRP3 ∆ exon 5 is inactive

To analyze the function of the major NLRP3 isoforms directly in primary human macrophages, we made use of splice-switching oligos (SSOs) to direct the splicing towards an increased percentage of NLRP3 ∆ exon 5. SSO morpholinos are nuclease-resistant DNA analogs^39^ designed to complementarily bind to the intron-exon boundary of pre-mRNA. By blocking the spliceosome’s access to a specific splice donor or acceptor site, the next possible respective site is typically used, resulting in AS of the targeted exon (Fig. 3f). Consistently, a SSO targeted to the intron 4 exon 5 boundary yielded near reversal of the ratio of full-length NLRP3 to NLRP3 ∆ exon 5 (Fig. 3g). hMDMs treated with a control morpholino responded to NLRP3 activation, while cells treated with the SSO released very low IL-1β amounts (Fig. 3h, left panel). Despite the expected variance in the LPS response of hMDMs isolated from different donors (data represent pooled data from several donors), the SSO did not inhibit the priming or secretion of TNF (Fig. 3h, right panel). Together, these data establish that splicing of NLRP3 occurs in human macrophages and suggest that NLRP3 ∆ exon 5 represents a loss-of-function isoform of NLRP3.

**Fig. 4:**
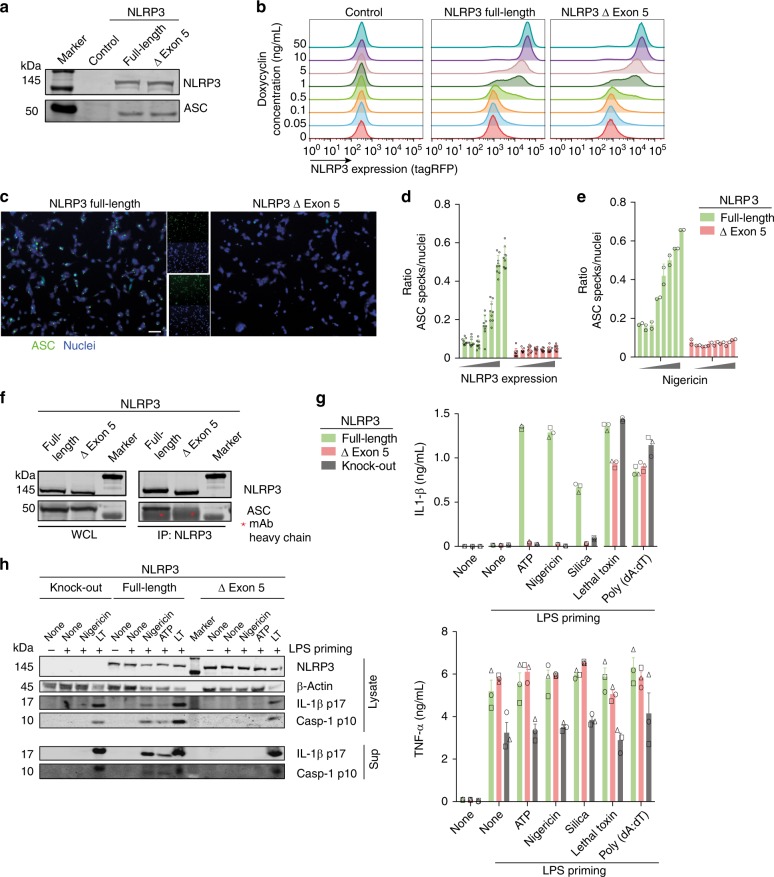
NLRP3 ∆ exon 5 is inactive. **a** Immunoblot expression control of NLRP3-tagRFP and ASC-mCerulean from HEK TREx Flp-In cells. **b** NLRP3-tagRFP was induced in HEK TREx with the respective concentrations of doxycycline. Expression levels were measured by FACS. **c** HEK TREx cells expressing inducible NLRP3-tagRFP and ASC-mCerulean were analyzed for ASC speck formation by fluorescence microscopy. Cell nuclei were counterstained with Draq5. Scale bar represents 50 μm. **d** Quantification of ASC speck formation after doxycycline induced NLRP3 overexpression (0–10 ng/mL). Mean and SD of nine frames per condition. Overlaid symbols represent single measurements. **e** Quantification of ASC speck formation after 2.5 h stimulation with nigericin (0–10 µM). Mean and SD of technical duplicates, nine frames per well, representative of three independent experiments. Overlaid symbols represent single measurements. **f** Co-immunprecipitation (IP) of ASC with NLRP3-tagRFP from HEK TREx Flp-In cells. NLRP3 was immunprecipitated using anti-tagRFP mAb. Asterisk indicates heavy band of IP mAb. Representative of two independent experiments. **g** and **h** NLRP3-deficient immortalized macrophages (iMos) were retrovirally reconstituted with either NLRP3 full-length or NLRP3 ∆ exon 5. **g** Cytokine secretion after priming with LPS (TNF) and activation of the NLRP3, NLRP1b, or AIM2 inflammasomes (IL-1β). Mean and SD of technical triplicates, representative of three independent experiments. Each individual data point from one experiment is plotted using a unique overlaid symbol shape. **h** Immunoblots of iMos after activation of the NLRP3 inflammasome (ATP, nigericin) or the NLRP1b inflammasome (lethal toxin). Blots are representative of two independent experiments. See also Supplementary Fig. 4. Source data are provided as a Source Data file

The relative high expression level of NLRP3 ∆ exon 5 suggests a deliberate isoform rather than splicing noise. To elucidate a potential function of the splice variant independent of the full-length variant, we made use of different model systems. HEK cells lack expression of NLRP3 inflammasome components and can thus be used as a minimalistic cellular environment to test the intrinsic activity of NLRP3 independent of most regulatory effectors. We reconstituted the respective NLRP3 isoforms and ASC as fluorescently tagged versions (encoded on a single plasmid) and established cell lines using Flp-In technology in HEK TREx cells (Fig. 4a). Since overexpression of NLRP3 is sufficient to cause activation in HEK cells, we made use of the tetracycline inducible expression system. Doxycycline dose-dependently induced overexpression of both isoforms of NLRP3 (Fig. 4b). However, overexpression led to activation of the full-length but not the ∆ exon 5 NLRP3 variant (Fig. 4c, d). Similarly, when these NLRP3 expressing HEK cells were activated using nigericin, we observed ASC speck formation only in cells expressing the full-length but not the ∆ exon 5 version of NLRP3 (Fig. 4e). We further tested whether the lack of response in the ∆ exon 5 NLRP3 variant is also observed at the level of ASC polymerization, or whether the interaction of ASC and NLRP3 is disturbed. Co-IPs of NLRP3 and ASC showed that the isoform ∆ exon 5 failed to interact with ASC, whereas full-length NLRP3 could co-immunoprecipitate ASC as expected (Fig. 4f).

To analyze the NLRP3 isoforms in the context of naturally inflammasome competent cells, we made use of NLRP3-deficient immortalized macrophages (iMos), which we virally reconstituted with either full-length NLRP3 or NLRP3 ∆ exon 5 (Supplementary Fig. 4a). Cells were primed with LPS and either treated with NLRP3 specific activators or as control with stimuli of the NLRP1 or AIM2 inflammasome. Only NLRP3 full-length and not NLRP3 ∆ exon 5 reconstituted cells were able to mount an NLRP3 specific inflammasome response (Fig. 4g), while all cell lines showed comparable amounts of TNF secretion after LPS priming and were fully capable to secrete IL-1β after activation of the NLRP1 or AIM2 inflammasome, excluding any general defects in the inflammasome pathway (Fig. 4g). Consistently, iMos reconstituted with NLRP3 ∆ exon 5 were neither capable of producing the mature form of IL-1β nor could they cleave pro-caspase-1 upon NLRP3 activation as shown by western blot (Fig. 4h). Likewise, IL-1β secretion after activation of the non-canonical inflammasome and after potassium-independent activation of NLRP3 could only be detected for NLRP3 full-length expressing cells (Supplementary Fig. 4b–d).

### Stochasticity of NLRP3 splicing

**Fig. 5:**
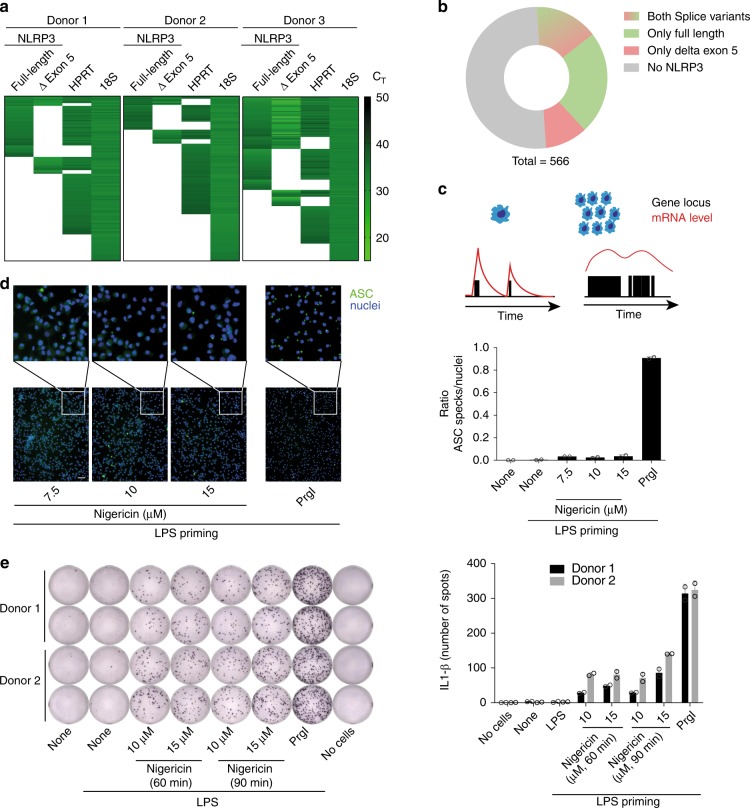
NLRP3 splicing is regulated on a single cell level. **a** Single PI-negative GM-CSF derived hMDMs were FACS-sorted into individual wells and lysed. RNA was reverse transcribed and NLRP3 full-length, NLRP3 ∆ exon 5 and HPRT encoding mRNAs were pre-amplified. Transcripts were detected with nested TaqMan assays. 187 to 192 individual cells per donor. **b** Quantification of the single cell NLRP3 splice pattern. Shown as the mean of three donors from **a**. **c** Scheme of the burst-kinetic of gene-expressions on single-cell level, resulting in oscillations of produced mRNA levels per gene. **d** Human monocyte-derived macrophages were analyzed for ASC speck formation by fluorescence microscopy after NLRP3 activation with nigericin and NLRC4 activation with bacterial product PrgI (both 1.5 h). Cell nuclei were counterstained with Draq5. Scale bar represents 50 μm. Five images per well were captured, plotted are means and SD of two replicate wells, representative of four individual experiments. Overlaid symbols represent single measurements. **e** IL-1β ELISpot assay of hMDMs after NLRP3 or NLRC4 inflammasome activation. Shown are two independent donors. Mean and SD of technical duplicates, two independent donors. Overlaid symbols represent single measurements. See also Supplementary Fig. 5. Source data are provided as a Source Data file

Splicing can be regulated on single cell level, resulting in sub-populations of cells expressing either one, several or neither isoform. We hypothesized, that AS of NLRP3 might result in a sub-pool of cells with different activation characteristics. We primed hMDMs with LPS to induce NLRP3 expression and analyzed NLRP3 full-length and NLRP3 ∆ exon 5 expression by single cell PCR. We analyzed 187 to 192 single cells of three independent donors for their expression of NLRP3 full-length, NLRP3 ∆ exon 5, HPRT, and 18S rRNA. Although a fraction of cells expressed both splice variants, the majority of cells expressed only one of the splice variants or did not show NLRP3 mRNA expression at all (Fig. 5a, b). Due to the intrinsic properties of gene expression on single cell level, underlying a burst kinetic, the detected expression level of a gene in a single cell cannot be interpreted as the transcriptome of bulk cells (Fig. 5c). Although the concentration of protein products is kept relatively stable, mRNA levels are highly fluctuating. Upon opening of a gene locus, the mRNA content is rapidly increased and slowly declines to a minimum followed by the next peak (Fig. 5c). Since these expression bursts are not synchronized in a pool of cells, bulk RNA shows a stable expression mean at all times, which can serve as a good indicator for the protein product. 18S rRNA can be considered as an exception, as it is expressed several magnitudes higher and the functional unit is the RNA and not a protein product and therefore kept stable. Therefore, we used 18S rRNA, instead of HPRT, as a measure to control for technical dropouts but did not calculate any relative expression levels (Fig. 5a, b). This analysis demonstrates that NLRP3 splicing is likely not deterministic as we detected cells expressing both NLRP3 full-length and NLRP3 ∆ exon 5 at the same time. Stochasticity in gene transcription influences variability of cell responses as single cells can behave differently depending on the expression level of any given gene. This analysis further demonstrates that there is large interindividual variability regarding expression of NLRP3 in single cells.

To assess whether the stochastic expression of NLRP3 might contribute to an adjustable response of cells to danger signals, we evaluated whether AS of NLRP3 can affect the number of cells responsive to NLRP3 triggers. We made use of two independent assays to analyze the activation of single cells after inflammasome activation in hMDMs. First, we evaluated the percentage of cells forming an inflammasome speck upon NLRP3 or NLRC4 activation (Fig. 5d). Surprisingly, and in contrast to mouse macrophages (Supplementary Fig. 5a), only a minor fraction of hMDMs responded to NLRP3 stimuli. In contrast, nearly all hMDMs responded on a NLRC4 trigger, showing that inflammasome activation is achievable in these cells. Noteworthy, a decent IL-1β response could still be detected after NLRP3 activation (Supplementary Fig. 5b). To rule out a suboptimal activation of the human cells by nigericin we tested increasing concentrations and prolonged activation times (Fig. 5d, Supplementary Fig. 5c). To test how many cells contribute to the IL-1β secretion, we performed ELISpot assays, which measure IL-1β release from single cells. Analogous to the ASC speck assays, only a minor fraction of hMDMs contributed to the IL-1β release downstream of NLRP3, while nearly all cells reacted upon triggering the NLRC4 inflammasome (Fig. 5e), providing evidence for the functional presence of all mutual inflammasome components. Together, these results suggest that the NLRP3 inflammasome in human macrophages is regulated on many levels, likely including the level of gene expression and AS.

### NLRP3 ∆ exon 5 lacks the interaction surface for NEK7

**Fig. 6:**
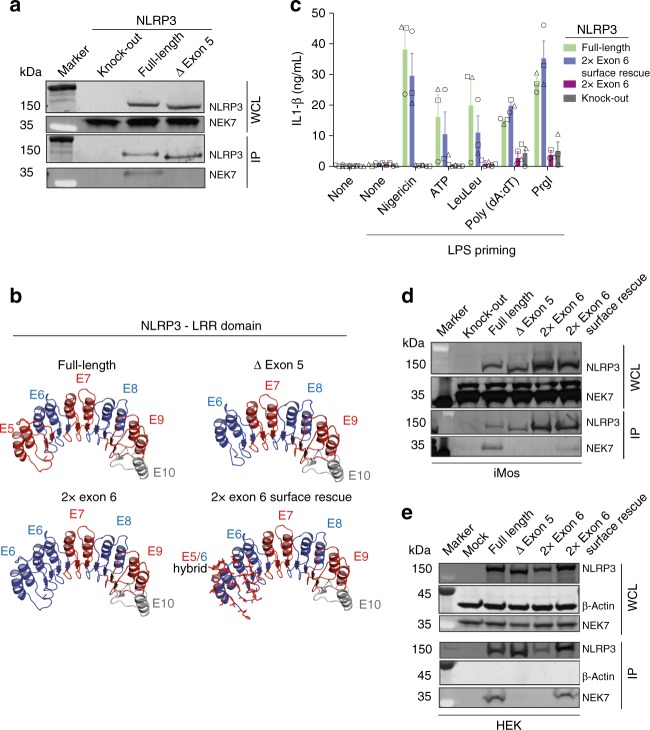
NLRP3 ∆ exon 5 lacks NEK7 interaction surface. **a** Co-immunprecipitation (IP) from iMos stably expressing the respective NLRP3-mCitrine variants. IP was performed in GFP-trap plates. **b** Models of the NLRP3 LRRs based on the crystal structure of human ribonuclease inhibitor. Shown are the LRR model structures of NLRP3 full-length and NLRP3 ∆ exon 5, as well as two artificially created hybrid LRRs: NLRP3 LRR lacking exon 5 but carrying a duplicate exon 6, and NLRP3 LRR carrying a duplicate exon 6 in which all surface amino acids of exon 5 were rescued. **c** NLRP3-deficient iMos were reconstituted with full-length or the hybrid NLRP3-mCitrine variants from **b**. IL-1β measured after priming with LPS and activation of the NLRP3, AIM2 or NLRC4 inflammasomes. Mean and SEM of three experiments. Each individual data point from one experiment is plotted using a unique overlaid symbol shape **d** Co-IP from iMos stably expressing the respective NLRP3-mCitrine variants. IP was performed in GFP-trap plates. Representative of two independent experiments. **e** Co-IP from HEK cells transiently transfected to express the respective NLRP3-mCitrine variants. IP was performed in GFP-trap plates. Representative of three independent experiments. See also Supplementary Fig. 6 and Supplementary Table 2. Source data are provided as a Source Data file

NEK7 binding to NLRP3 was demonstrated as a prerequisite for NLRP3 activation and the NLRP3 binding site was suggested to be located within the LRR^18^, yet the exact area of NEK7 binding on NLRP3 has not been identified. Therefore, we performed interaction studies between the different NLRP3 isoforms and NEK7 using iMo cell lines. Co-immunoprecipitation of NLRP3 demonstrated an interaction of NLRP3 with endogenous NEK7 for the full-length isoform but not for the NLRP3 ∆ exon 5 isoform (Fig. 6a, Supplementary Fig. 6a).

Next, we wondered whether the NLRP3 ∆ exon 5 isoform might be inactive as a result of a shorter LRR. An alternative hypothesis was that the amino acids encoded by exon 5 are specifically needed for the activation. To address these questions we created an artificial NLRP3 version encoding a duplication of exon 6 but no exon 5, so that the total LRR length was conserved. We further created an NLRP3 version, in which exon 6 was duplicated, but in the duplicated exon 6 all surface defining amino acids were revised to the exon 5 amino acids (Fig. 6b). Due to the high level of conservation of the LRR exons, the overall physico-chemical characteristics of the hybrid isoforms is similar to the wildtype (wt) isoforms (Supplementary Table 2). Stable iMo cell lines were created as before and the cells were primed with LPS followed by NLRP3, AIM2, or NLRC4 activation. Although AIM2 and NLRC4 activation resulted in comparable levels of IL-1β secretion across all tested cell lines, IL-1β secretion could not be observed after NLRP3 activation in the NLRP3-deficient cells and in those reconstituted with the doubled exon 6. However, iMos expressing the NLRP3 variant with a doubled exon 6 but all surface amino acids of exon 5 in the first two repeats, were fully active (Fig. 6c). All cells were properly primed as evidenced by comparable levels of TNF secretion (Supplementary Fig. 6b).

Again, we performed co-IPs and tested the ability of all NLRP3 variants to interact with NEK7. While the duplicate exon 6 could not interact with NEK7, the NLRP3 variant with a duplicated exon 6 but a surface rescued to exon 5 showed a partial rescue of the interaction with NLRP3 in iMos (Fig. 6d). Although this partial rescue seems to be sufficient to allow for full inflammasome activation, we tested whether the interaction is fully recovered in a system in which human NLRP3 interacts with human NEK7. Indeed, performing the same experiment after transient transfection of HEK cells with NLRP3 variants demonstrated a full rescue of the NLRP3-NEK7 interaction as soon as all exon 5 surface amino acids were expressed (Fig. 6e). Taken together, our data show that NEK7 binds to the LRRs encoded by exon 5 of NLRP3.

## Discussion

Except for the highly variable receptors involved in adaptive immune response, every nucleated cell carries the same genetic information. Genes encode for proteins and regulatory RNAs and their coordinated expression determines cell identity and function. Functional diversity of proteins can be considerably increased by AS, which is much more abundant in higher than in lower eukaryotes. Although different species such as nematodes, mice, and humans carry approximately the same amount of protein coding genes (~20,000), they express about 50,000, 100,000, and 200,000 different isoforms, respectively. Accordingly, the increased complexity is mostly achieved by a more diverse AS pattern, not by more coding genes. As proteins are key molecules that coordinate nearly all cellular functions, AS can dramatically influence cell identity and cell specific functions^40^. Although the higher prevalence of AS in humans compared to mice is known, a gene-specific prediction of its impact is difficult. It is conceivable that the rather detrimental NLRP3 activity associated with higher age of humans requires a higher level of regulation than in lower species, where communicable diseases represent the higher health thread. This would be in line with other regulatory factors like pyrin- or CARD-only proteins, shaping the human, but not murine inflammasome response^10^.

Around 500 million years ago, diversification of the adaptive immune system was achieved with the evolution of two different recombinatorial approaches in vertebrates. In jawed vertebrates the protein diversity of the immunoglobulin domain-based T and B cell antigen receptors is achieved through somatic rearrangement of V(D)J gene segments and hypermutation, while in jawless vertebrates, somatic recombination of LRR sequences evolved to create diversity in VLRs^34^. Hence, alternative LRR splicing could represent (by the use of different means than VLRs) a further level of increased functional diversity in the innate immune system. Of note, the here detected exons, mostly encoding for blocks of 24 or 28/29 aa, represent LRR modules of self-compatible building blocks to create well folded protein structures when repeatedly stacked^36^.

As in NLRs, domain boundaries and exon junctions in the majority of genes show consistent strong correlations, which further increases from invertebrates towards higher vertebrates^41^. This not only allows for genomic exon re-shuffling^42^, but also facilitates recombination or deletion of functional units by AS. Of interest, in the plant immune system, AS of resistance genes has evolved to regulate the immune response to pathogens and stress^43,44^. Given the structural and functional homology of plant resistance proteins and vertebrate NLRs, our findings solidify the importance of this mechanism. Interestingly, inflammasome responsiveness can also be modulated via differential splicing of ASC, a mechanism tuning the responsiveness of cells for several different inflammasome sensors. ASC-b lacks the flexible inter domain linker, reducing the efficiency of ASC speck formation, and ASC-c lacks most of the PYD and inhibits IL-1β maturation via a competitive mechanism^45^. Furthermore, human NLRP3 mRNA can be alternatively polyadenylated, resulting in a shortened 3′UTR, which lacks binding site for the negative regulators miRNA-223 and tristetraprolin^46^.

Although long recognized, the role for alternative splicing among the sensor proteins was not well understood. We noted multiple splice variants in our initial description of NLRP3^47^. NLRP3 isoforms lacking exon 5, or 5/7 were later tested for their NF-kB suppressive properties, with no effect identified^48^. By performing single-cell analysis of NLRP3 splicing of exon 5, we have identified that NLRP3 splicing is likely not deterministic as we could identify cells expressing both the full-length NLRP3 isoform as well as the isoform lacking exon 5. Although this analysis cannot rule out that individual cells might be in the process of shifting from expressing only one splice form to expressing another splice form, it is tempting to speculate that AS of NLRP3 contributes to the stochastic nature of NLRP3 activatability. The stochasticity of the inflammasome response causes stimulated cells to exhibit large variability in their response to danger signals. As the NLRP3 inflammasome is highly regulated by many feed-forward as well as negative feed-back loops, stochasticity would allow for fine-tuning of a graded response that is adjusted to the type and magnitude of danger molecules recognized by NLRP3. At the same time, different activation thresholds of NLRP3 could be important to prevent a coordinated pyroptotic cell death of macrophages during the presence of danger signals, which could subject the host to increased susceptibility for infections. If the NLRP3 LRR acts as a self-inhibiting domain, as shown for NLRC4^35^, NEK7 might stabilize an activatable state rather than causing activation. Indeed, a nonsense mutation in NLRP3 exon 4 (R554X) results in the complete loss of the LRR and the patient presents an inflammatory FMF/FCAS-like phenotype^49^, suggesting that self-inhibition of NLRP3 is lost. Moreover, it was shown that an artificial NLRP3 variant lacking the LRR could still be activated^50^.

Our bioinformatics analysis showed that NLR proteins share the modularity of exons encoding for LRRs with several protein families that carry short repetitive LRR encoding exons of the RI and S/T class of LRRs, including Slit proteins involved in neuronal axon guidance. A large heterogeneity of Slit mRNA could be identified, and their binding properties are suggested to depend on varying number of LRR repeat units^51,52^. LRRC37 was restricted to the testis, but rapidly evolved in the hominid lineage with higher levels of expression in the cerebellum and thymus and increasing diversity of alternative splice forms^53^. Hence, AS of LRR encoding exons appears to be a mechanism to create functional diversity not only in the immune system.

General deficiencies in the splicing machinery are not compatible with life and gene-specific alternations in AS can result in diseases and organ failures^54^. Cancer, cardiovascular diseases, diabetes and neurological diseases have each been correlated with specific changes in AS^54^. Consequently, several approaches have been developed to correct disease-associated aberrant splicing or to correct the reading frame after exon duplications or deletions. Two SSO drugs have been recently approved to treat Duchenne muscular dystrophy and spinal muscular atrophy^55,56^. Our studies suggest that using SSOs to induce the skipping of NLRP3 exon 5 in NLRP3-driven diseases might hold therapeutic potential in diseases associated with increased activity of NLRP3.

## Methods

### Isolation of primary cells, plasmids, and cell lines

Primary human and pig cells were isolated from blood using ficoll gradient centrifugation to obtain PBMCs. Human monocytes were isolated from PBMCs with CD14 microbeads (Milteny) according to the manufacture’s instruction. Macrophages were differentiated from monocytes by incubation with rhGM-CSF (500 U/mL) for 3 days. M1 macrophages were further differentiated for three additional days with IFNγ (200 U/mL) and GM-CSF (500 U/mL), M2 macrophages with IL-4 (1000 U/mL) and GM-CSF (500 U/mL). Medium for all human cells is complete RPMI with 10% FCS, Glutamax, Na-Pyruvate and Penicillin/Streptomycin (all three 100x stocks from Thermo-Fischer).

Mouse bone marrow-derived macrophages (BMDMs) were obtained by culturing bone marrow cells from WT C57BL/6 mice in DMEM supplemented with 10% fetal calf serum (FCS) and 20% L929 conditioned medium. Immortalized NLRP3-deficient mouse macrophages (iMos) were previously described^57^. HEK cells and iMos were cultured in DMEM with 10% FCS and 100 U/mL penicillin/streptomycin. The HEK293-FlpIn TREx cell line (Invitrogen) was used to create cell lines with a single insertion of a single copy of the genes of interest. NLRP3-deficient iMos were retrovirally transduced with constructs for the indicated NLRP3 variants fused C-terminally to an mCitrine-tag. After retroviral transduction, cells were flow cytometrically sorted to similar levels of expression (Supplementary Fig. 7).

All plasmids were obtained using standard cloning techniques. The protein sequence of NLRP3 2x Exon 6 surface rescue is as shown in Supplementary Note 1.

### RNA isolation, NLRP3 LRR PCR and qPCR

RNA was isolated using the RNeasy kit from Quiagen, according to the manufactures instructions. RNA (250–1000 ng) were used to reverse-transcribe cDNA using an oligo dT(18) primer and SuperScript III reverse transcriptase (Invitrogen).

Whole LRR PCR was performed on cDNA with EconoTaq PLUS GREEN 2x mastermix from Lucigen using primers outside of the highly conserved LRR region. To minimize any PCR bias towards possible smaller isoforms the elongation phase of each cycle was set to 3 min. The primers used were the following: for human, P1 and P2;, for mouse, P3 and P4; for pig, P5 and P6.

cDNA abundance was analyzed using Maxima, SYBR Green/ROX qPCR Master Mix from ThermoFischer on a QuantStudio6 cycler. The primers used were the following: for HPRT, P7 and P8; for NLRP3 Exon 4–5, P9; for NLRP3 exon 5–6, P10; for NLRP3 exon 4–6, P11; for NLRP3 exon 6–7, P12; for NLRP3 exon 6–8, P13; and for NLRP3 exon 6, P14. All primer sequences are shown in Supplementary Table 3.

### Single-cell PCR

hMDMs were primed with 2 ng/mL LPS for 3 h. Single cells were sorted into 96-well plates. Cells were lysed, RNA was reverse transcribed and preamplification for NLRP3 variants and HPRT was performed for 20 cycles (AmpliTaq Gold, Thermofischer), before cDNA was split up for the detection of different transcripts by TaqMan PCR. 18S RNA was not pre-amplified. Primers for preamplification were: for NLRP3, P15 and P16; for NLRP3 ∆exon 5, P17 and P18; for HPRT, P19 and P20. Taqman assays were from Thermo Fischer (HPRT HS02800695, 18S HS99999901_s1) or self-designed (labeled with 6-carboxyfluorescein (6-FAM) on their 5′ end and a non-fluorescent black hole quencher (BHQ-1) on their 3′-end, Metabion) NLRP3 (primers P21 and P22, probe: aacgctccagcatcctggctgtaaca) NLRP3 ∆ exon 5 (primers P23 and P24, probe: ttctcatgggttggggc). Reaction mix was GoTaq Probe qPCR Master mix (Promega). All primer sequences are shown in Supplementary Table 3.

### Sequencing of isoforms

The lower band lacking at least one exon was cut from the gel, the PCR fragment was isolated and blunt ligated into a plasmid (CloneJET PCR Cloning Kit, ThermoFischer), Bacteria were transformed and single clones were picked. Minipreps of single clones were performed and DNA was sent for sanger sequencing at GATC^58^.

### Stimulation conditions

iMos were primed with 200 ng/mL LPS (Ultrapure LPS, Invivogen) for 3 h. Inflammasome activation was performed with 10 µM nigericin (Life Technologies) and 5 mM ATP (Sigma) for 1 h or with 1 mg/mL Silica (US-Silica), 1 mM LeuLeuoMe (ChemImpex), lethal toxin (1 µg/mL lethal factor (List Biologicals), 1 µg/mL, protective antigen (List Biologicals), poly(dA:dT) (20 ng/mL + 0.5% lipofectamin 2000), LFn-PrgI (4 µg/mL (M. Geyer) + protective antigen 1 µg/mL) for 3 to 4 h, 12 or 25 µg/mL PGN from *S. Aureus* (Invivogen) for 20 h, 20 µg/mL R837 (Invivogen) for 1.5 h, cytosolic LPS (Cholera toxin subunit B (CTB) (20 μg/mL + 2 μg/mL LPS), or with DOTAP (3.75 μg/mL + 750 ng/mL LPS)) for 16 h.

iMos for activated NLRP3-NEK7 Co-IP were primed with 200 ng/mL LPS for 2 h. CRID3 (Pfizer) was added for the last h and caspase inhibitor YVAD was added 20 min before nigericin to prevent pyroptosis. Cells were activated with 10 µM nigericin for 20 min.

hMDMs were primed with 2 ng/mL LPS for 3 h and activated with 10 µM nigericin for 1 h or LFn-PrgI (3 ng/mL) + protective antigen 1 µg/mL) for 1 h.

### Cytokine measurement

Secreted cytokines were measured by ELISA or HTRF according to manufactures instructions (ELISA kits for mouse IL-1β and mouse TNFα from R&D Systems, HTRF kits for mouse and human IL-1β and TNFα from Cisbio).

ELISpot assays were performed using the hIL-1β kit from abcam according to the manufacturer’s recommendations. To assure enough distance between single cells and to allow for single spot discrimination, 300 hMDMs were seeded per 96 well.

### Immunprecipitation

Cells were washed with PBS and lysed in RIPA buffer or Co-IP lysis buffer (50 mM Tris pH 7.8, 50 mM NaCl, 0.1% NP-40, 10 % Glycerol, cOmplete protease inhibitor and PhosSTOP (Roche), PMSF (0.2 mM). Nuclei were removed by centrifugation (10 min, 1000×*g*) and protein concentration was normalized after measurement by bichinonic acid assay (BCA; Thermo Fisher Scientific).

Endogenous NLRP3 was immunprecipitated using an NLRP3 antibody (D4D8T, Cell signaling) bound to protein G Dynabeads (Life Technologies). IP was performed for 4 h at 4 °C. Samples were denatured with LDS buffer supplemented with reducing agent (Thermo Fisher) for 10 min at 85 °C.

Co-immunprecipitation of NLRP3-mCitrine was perfomed in GFP-trap plates (Chromotec) for 2.5 h at 4 °C shaking or using a tRFP antibody (AB233, evrogen) coupled to protein G Dynabeads. Samples were denatured as described before.

### Sample preparation and Immunoblots

Samples were prepared and immunoblots performed as described before^58^. In brief, cells were washed with PBS and lysed with NP-40 buffer (20 mM Tris-HCl, pH 7.4, 150 mM NaCl, 1 mM EDTA, 1% Nonidet P-40, 10% glycerol, cOmplete protease, and PhosSTOP (Roche) inhibitor). Nuclei were removed by centrifugation (10 min, 1000×*g*) and protein concentration was normalized after measurement with a bichinonic acid assay (BCA; Thermo Fisher Scientific). Samples were reduced and denatured by adding NuPAGE LDS Sample Buffer (4×) and NuPAGE Sample Reducing Agent (10×) (both from Thermo Fisher) and heating at 85 °C for 10 min. Proteins were separated by 4–12% SDS-PAGE in precast gels (Novex; Invitrogen) with MOPS buffer (Novex; Invitrogen). Proteins were transferred onto Immobilon-FL PVDF membranes (Millipore) and nonspecific binding was blocked with 3% BSA in Tris-buffered saline for 1 h, followed by overnight incubation with specific primary antibodies in 3% BSA in Tris-buffered saline with 0.1% Tween-20.

The following antibodies were used: NLRP3 (cryo2, AG-20B-0014-C100, Adipogen; D4D8T, 151015, Cell Signaling) both 1:2000; β-actin (Licor) mouse (926–42212) or rabbit (926–42210), both 1:1000; caspase-1 p10 (M20, sc-514, SantaCruz) 1:200; IL-1β p17 (detection AB from R&D mouse ELISA Kit no. 840169) 1:1000; NEK7 (EPR4900, ab133514, Abcam) 1:500–1:2000; ASC (AL177, 210–905-C100, Adipogen) 1:1000; secondary antibodies (coupled to IRDye 800CW or IRDye 680RD LI-COR Biosciences) 1:25000.

### Splice-switching oligos

Morpholinos and Endo-porter were obtained from gene-tools.com. Day 3 differentiated hMDMs were seeded with GM-CSF (250 U/mL) in complete medium and were allowed to settle down before transfection. Morpholinos have been heated up to 65 °C before use to recover full activity. Morpholinos and Endo-porter have been premixed and were carefully added to the cells to a final concentration of 6 µM each. Cells were LPS primed at 39 h after transfection.

### Structural alignments and modeling of the NLRP3 LRR

Structural alignment of LRRs was performed using the RI class (LxxLxLxx(N/C)xLxxxgoxxLxxoLxzxxxx) or S/T class (LxxLxLxxNxLxxLpxxoFxzxLxx) consensus sequence.

Structural modeling was performed using SwissModel. The crystal structure of human ribonuclease inhibitor (PDB: 2q4g) was used as a template.

### ASC Speck analysis

HEK293-FlpIn TREx cells were treated overnight with the indicated doses of doxycycline or stimulated with 10 µM and respective lower doses of nigericin for 2.5 h. hMDMS were primed with LPS. 30 min before activation, the caspase-1 inhibitor VX-765 was added at a final concentration of 10 µM before cells were activated for 1.5 h. Caspase-1 deficient iMos were primed with LPS and activated with nigericin.

HEK293-FlpIn TREx cells were fixed with 2% formaldhyde and nuclei were stained with Draq5 (eBioscience, 1:2000) for 15 min at RT. hMDMs were fixed with 2% formaldehyde and lysed with Triton X-100 containing buffer. ASC was stained using an AF488 or 647 labeled ASC antibody (TMS-1) and nuclei were counterstained with Draq5 or DAPI. iMos were fixed with 4% formaldehyde and stained for ASC (2EI-7, F(ab’)2-AF647). Nuclei were counterstained using HOECHST. Cells were imaged using a Zeiss Observer.Z1 epifluorescence microscope. Images were quantified using Fiji^59^ or CellProfiler^60^ software.

### FACS analysis of NLRP3 expression

HEK293-FlpIn TREx NLRP3 cell lines were treated overnight with the indicated doses of doxycycline. The next morning HEKs were harvested and analyzed for their NLRP3-tagRFP expression levels. iMos were harvested without pre-treatment and analyzed for NLRP3-mCitrine expression.

### In silico approaches

Spliceport (http://spliceport.cbcb.umd.edu/) was used to analyze the strength of the splice donor and acceptor sites of human NLRP3^37^. The number of exonic splice enhancer sites within the LRR exons of human NLRP3 was assessed with RescueESE (http://hollywood.mit.edu/burgelab/rescue-ese/)^61^.

Physico-chemical characteristics of the (artificial) splice variants were calculated using the ProtParam online tool (https://web.expasy.org/protparam/).

### LRR exon structure analysis

LRR annotations for human proteins were performed using the ensmbldb Bioconductor Package^62^. All known canonical transcripts as defined by UCSC (GRCh38) were queried for LRR domains annotated by the SMART database, namely: SM00369, SM00370, SM00364, SM00367, SM00368, and SM00365.

All exons spanning LRR domains were extracted for further analysis: exons were divided into frame-preserving (divisible by 3) or frame-shifting and plotted for their length distribution. Genes contributing to the major peaks within the exon length distribution plot (69–75, 81–87, 141–147, and 168–174 bp) were identified and used to create a phylogenetic tree: amino-acid sequences were aligned using MUSCLE^63^ and the dist.ml function from the phangorn Bioconductor package^64^ was used with default settings to create a distance matrix that was then used to estimate an un-rooted phylogenetic tree using the Neighbor Joining clustering method.

### RNASeq

hMDM were primed for 3 h with 2 ng/mL LPS. RNA was isolated and RNA integrity was checked for every sample before library preparation using a RNA ScreenTape on a Tapestation. RNA content of samples was quantified using a Qubit device.

Total RNA of 2 µg were used as input material. The RNA Seq library was generated using ‘SENSE mRNA-Seq Library Prep Kit V2’ (Lexogen) according to the manufacturer’s recommendation with the following details: for reverse transcription and ligation the RTL buffer was used to generate rather bigger fragments. To further adjust the size of the library fragments, during the purification after second strand synthesis 14 µl PB and 2 µl PS were used. Before library amplification and adapter attachment, a test amplification was performed. The library was amplified over 12 cycles using the i7 index primers 7001, 7002, 7004, 7005, and 7007 to maintain the best possible color-balance during the first cycles of RNA Seq.

The fragment size of the generated library was controlled using a Tapestation D1000 Screen Tape.

RNA sequencing was performed on an Illumine HiSeq high output flow cell using V4 chemistry in the Next Generation Sequencing (NGS) Core Facility of the Medical Faculty of the University of Bonn. We aimed for a sequencing depth of  200 M reads per sample (2×125 bp). RNA libraries from 5 donors were pooled and distributed on 4 lanes.

### RNA sequencing analysis

Sequencing reads were aligned to the human genome (build GRCh38/hg38) using STAR aligner^65^ with default settings and transcript annotations from Ensembl GRCh38.90: > STAR --runThreadN 3 --genomeDir /genome/human/staridx_primary/ --sjdbGTFfile /annotation/Homo_sapiens.GRCh38.90.chr.gtf --readFilesIn Donor*_1.fastq Donor*_2.fq --outSAMtype BAM SortedByCoordinate

Transcript abundances for all samples were quantified using Kallisto^66^ with default settings >kallisto quant -i Homo_sapiens.GRCh38.cdna.all.gene.kidx -o $outdir -t 3 Donor*_1.fastq Donor*_2.fq

and then summarized to gene level abundances using the tximport Bioconductor Package^67^.

Sashimi plots were generated using the sashimi_plot utility from the MISO software^68^, and exon skipping events were quantified using MISO “exon-centric” percentage-spliced-in analysis. Soft-clipped adapter sequence was removed from all STAR aligned samples and reads were subsequently trimmed to a uniform length of 91 bases per read, using in-house developed scripts. MISO was run with the following command: > miso --run./index_dir/./bams/Donor*.sorted.bam --output-dir miso_out --read-len 91.

### Statistical analysis

Statistical analysis was performed using GraphPad Prism8, as indicated in figure legends.

### Ethics

Human primary cells were extracted from blood concentrates provided by the blood donation service of the University Clinics Bonn (ERC Ethikantrag Lfd. Nr. 184/16 “Activation and regulation of Inflammasomes (InflammAct)”. This study has complied with all relevant ethical regulations for animal testing and research.

### Reporting summary

Further information on research design is available in the Nature Research Reporting Summary linked to this article.

## Supplementary information

Supplementary Information

Reporting Summary

Source Data

## Data Availability

The source data underlying Figs. 1c, d, 1i, 2a, 3b–e, 3g, h, 4a, 4d–h, 5a, b, 5d, and 6a, 6c–e, and Supplementary Figs. 3,a–c, 3,e, 4b–d, 5a–c and 6a, b are provided as a Source Data file. RNAseq data used in Fig. 2 and Supplementary Fig. 2 have been deposited in the NCBI GEO database under accession number GSE117206. Other data are available from the corresponding author upon reasonable request.
