# Supplementary material for: Alternative splicing regulates stochastic NLRP3 activity
Source: Nat Commun. 2019 Jul 19;10:3238. doi: 10.1038/s41467-019-11076-1 (PMC6642158; doi:10.1038/s41467-019-11076-1)
Supplement: Supplementary file 1 — Supplementary Information [file 41467_2019_11076_MOESM1_ESM.pdf]

Alternative splicing regulates stochastic NLRP3 activity

Hoss *et al.*

Supplementary Figures

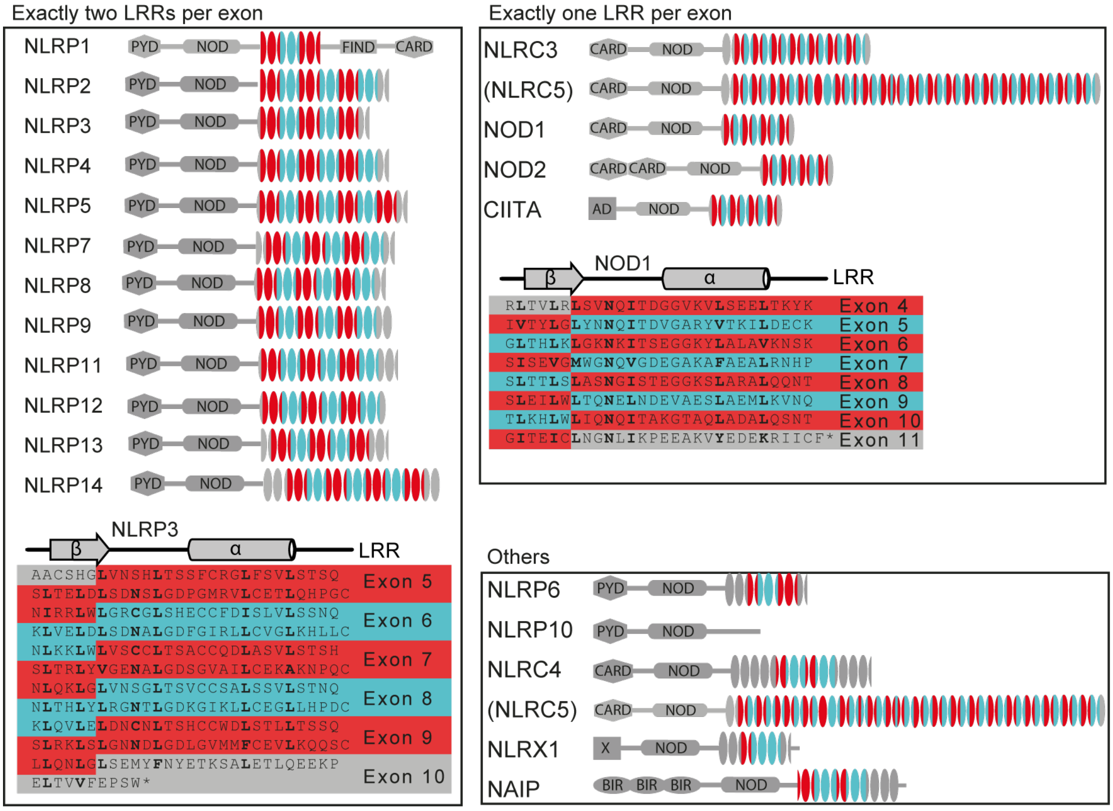

Supplementary Fig. 1
Most NLRs can be assigned into two groups according to their exon distribution within the LRR: Those in which each exon encodes two repeats (as in NLRP3) and those in which each exon encodes one repeat (as in NOD1). Structural alignments show the conserved exon-exon junction at the end of the β-sheet for both groups.

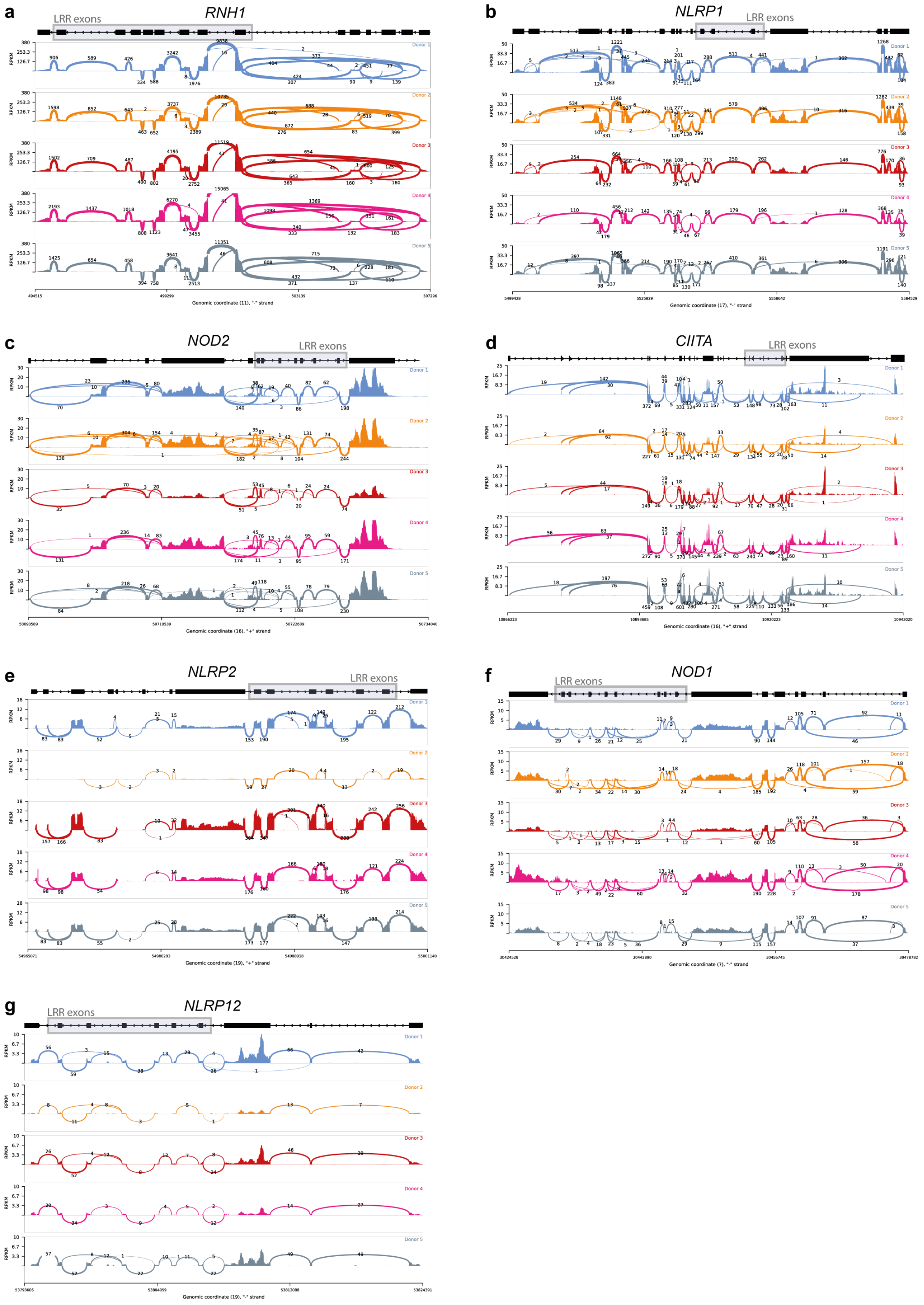

Supplementary Fig. 2
Sashimi plots as in Fig. 2b for **a** RNH1, **b** NLRP1, **c** NOD2, **d** CIITA, **e** NLRP2, **f** NOD1, and **g** NLRP12.

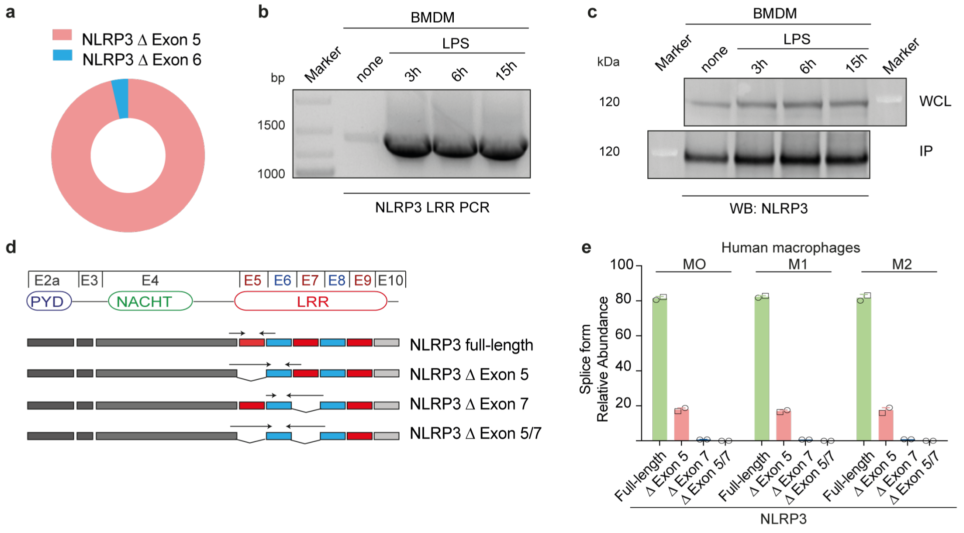

Supplementary Fig. 3

a The lower band of PCR reactions (as in Fig. 3b) after LPS priming was isolated and 28 clones were sequenced to determine, which exon is mostly alternatively spliced. **b** PCR of NLRP3 LRR on cDNA from mouse BMDMs after different timepoints of LPS priming. Representative of 3 independent mice. **c** Immunoblot of mouse NLRP3 from BMDMs. Either whole cell lysates or NLRP3 immunoprecipitates, using mAb against the NLRP3 NACHT domain, were immunostained with a mAb against the PYD to ensure NLRP3 specificity. Representative of 3 independent mice. **d** Scheme of the different qPCR primers targeted on the respective exon-exon boundaries for the annotated NLRP3 isoforms. **e** Relative abundance of the tested isoforms of NLRP3 in differentially polarized human macrophages. Values have been calculated as fraction of the overall NLRP3 after correction for primer efficiency. Mean and SD of 2 donors. Each individual data point from one donor is plotted using a unique overlaid symbol shape. Source data are provided as a Source Data file.

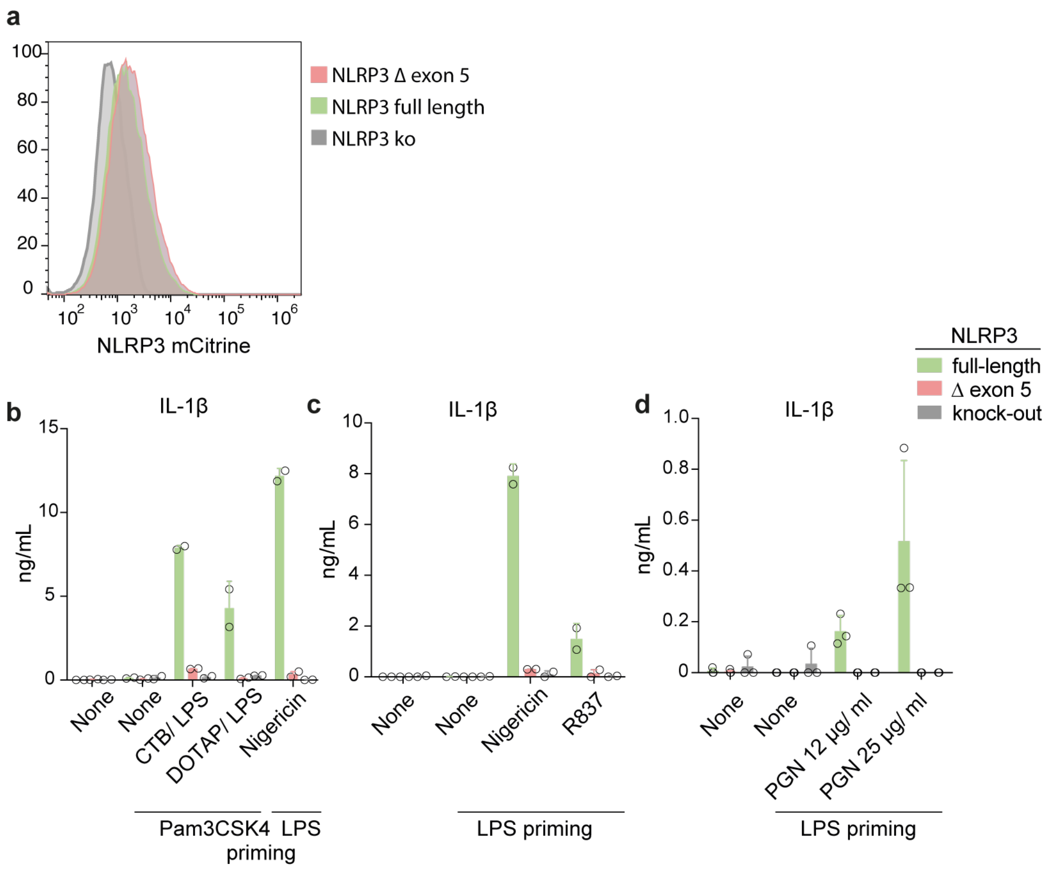

Supplementary Fig. 4

a Flow cytometric expression control of NLRP3-deficient iMos retrovirally reconstituted with NLRP3-mCitrine. **b** IL-1β secretion after Pam3CSK4 priming and non-canonical inflammasome activation. Mean and SD of technical duplicates representative of 2 independent experiments. **c** and **d** IL-1β secretion after potassium-independent NLRP3 inflammasome activation via R837 and PGN. **c** Mean and SD of 2 independent experiments. Individual data points from each experiment are plotted using the same overlaid symbol shape. **d** Mean and SD of technical triplicates. Overlaid symbols represent single measurements. Source data are provided as a Source Data file.

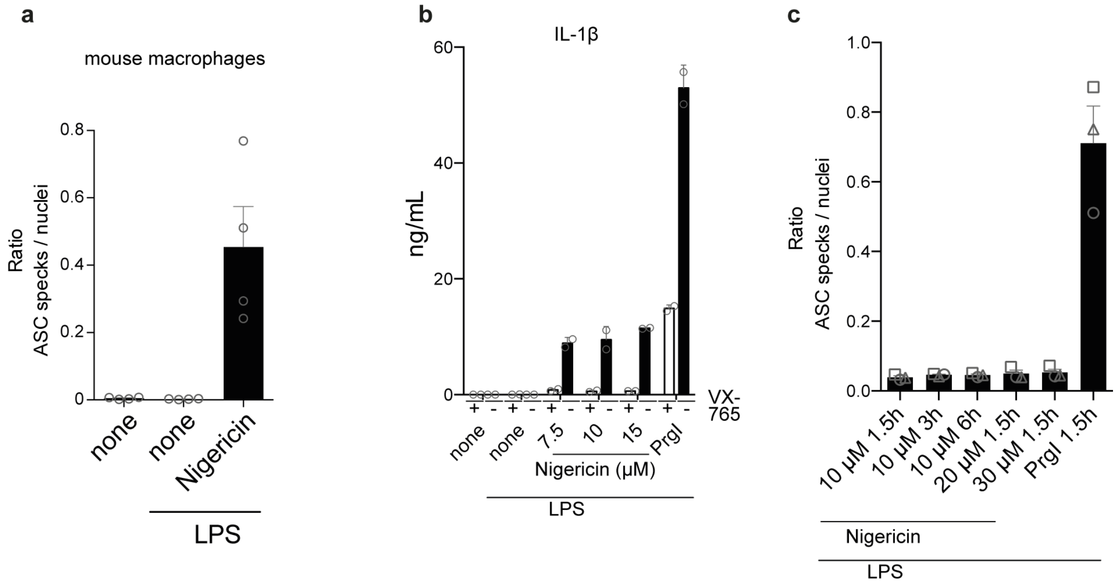

Supplementary Fig. 5

a ASC speck analysis of iMos after NLRP3 activation with nigericin. Mean and SEM of 4 independent experiments. Individual data points from each experiment are plotted using the same overlaid symbol shape. **b** Secretion of IL-1β measured in parallel to 5d with and without caspase-1 inhibtor VX-765 treatment (speck assays were VX-765 treated to prevent pyroptosis). **c** ASC speck analysis of hMDMs after activation as indicated. Mean and SEM of 3 independent donors. Each individual data point from one donor is plotted using a unique overlaid symbol shape. Source data are provided as a Source Data file.

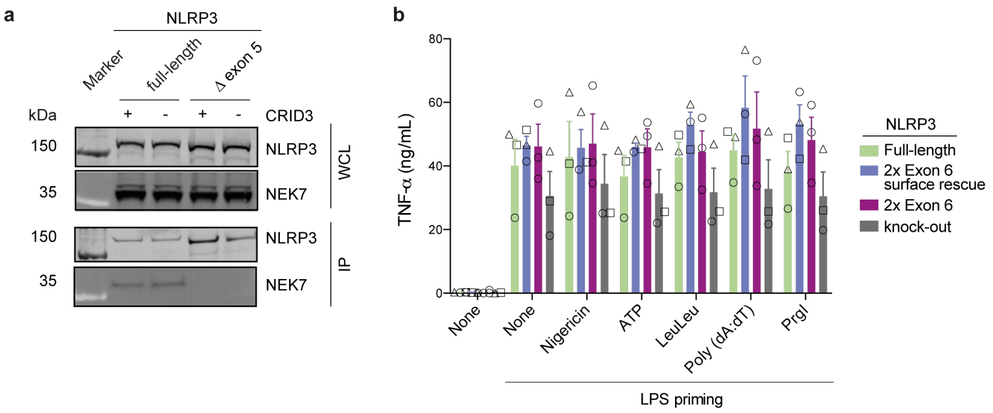

Supplementary Fig. 6

a as Fig. 6A, but from LPS-primed and nigericin-activated iMos with and without treatment with the NLRP3 inhibitor CRID3. Representative of 2 independent experiments **b** TNF secretion after LPS priming in Fig. 6b. Shown as Mean and SEM of 3 independent experiments. Individual data points from each experiment are plotted using the same overlaid symbol shape. Source data are provided as a Source Data file.

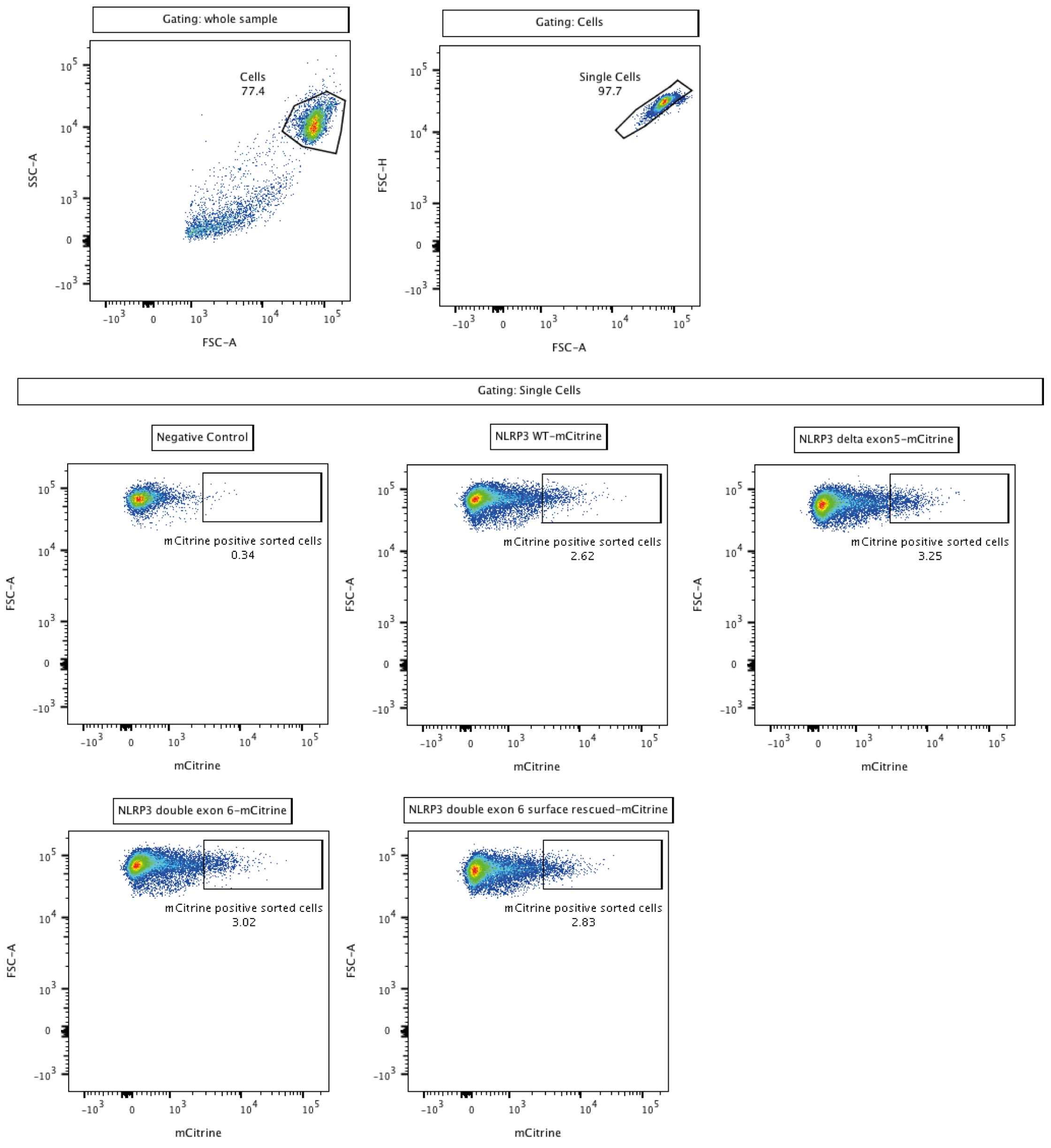

Supplementary Fig. 7

Gating strategy for the sorting of NLRP3 KO iMos retrovirally reconstituted with either NLRP3 WT-mCitrine, NLRP3 Δ Exon 5-mCitrine, NLRP3 2x Exon 6-mCitrine, or NLRP3 2x Exon 6 surface rescue-mCitrine that were used in Fig. 4 f-h, Fig. 6 a, c and d, Supplementary Fig. 4 and Supplementary Fig. 6a and b.

Fig. 3b

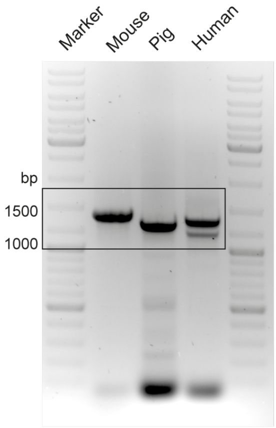

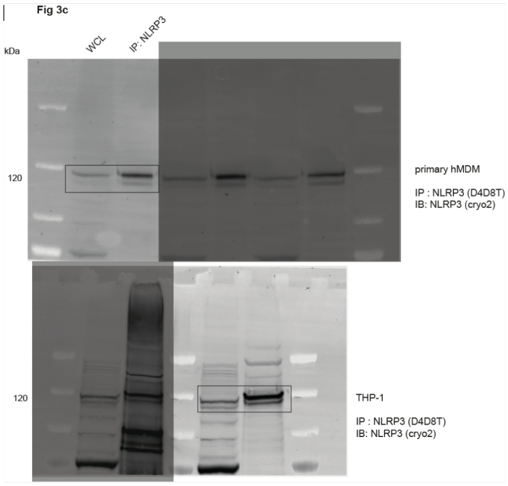

Fig. S3a

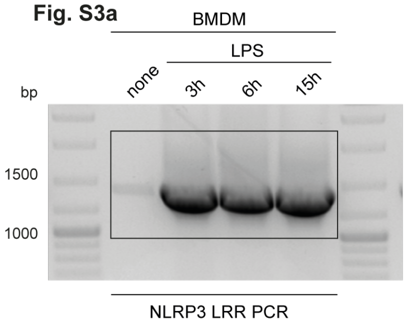

Fig. S3b

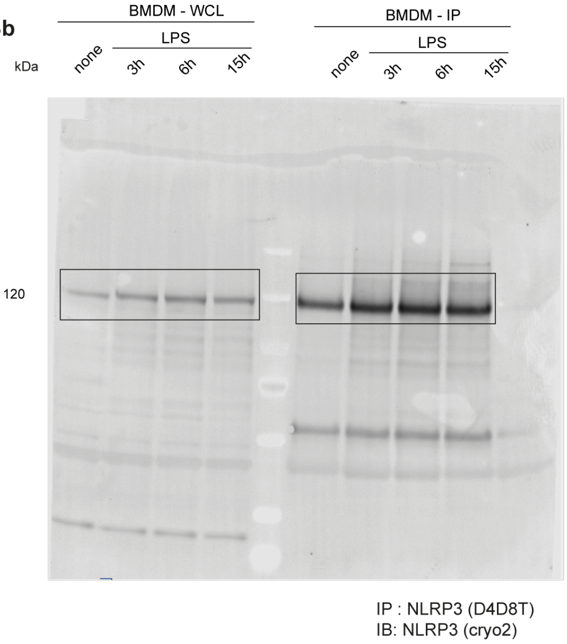

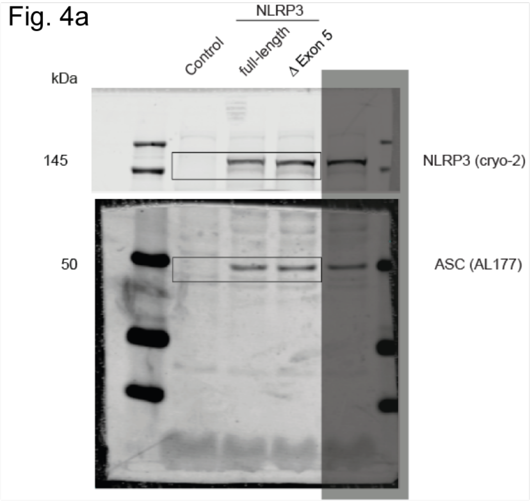
Fig. 4a
NLRP3
Control
full-length
Δ Exon 5
kDa
145
50
NLRP3 (cryo-2)
ASC (AL177)

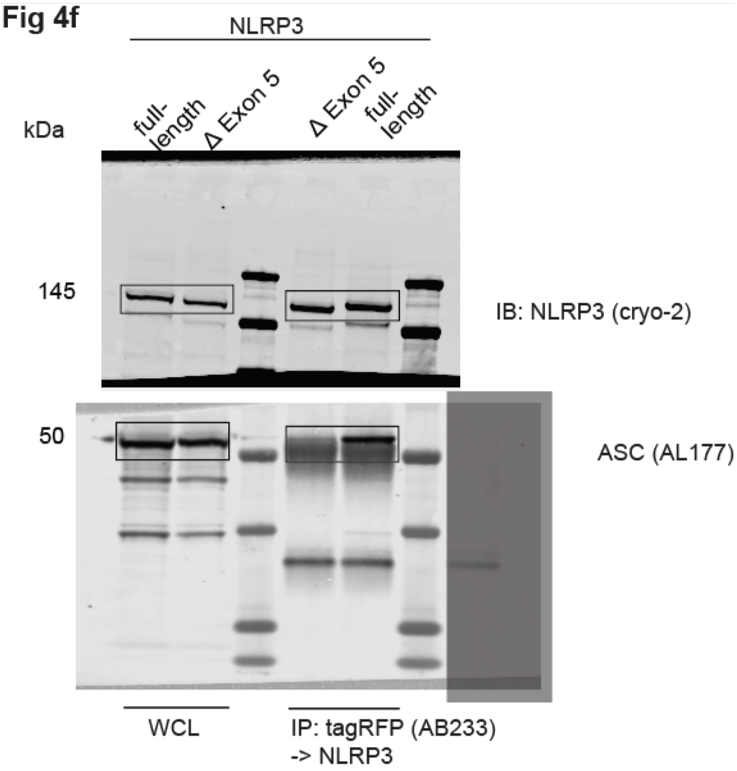
Fig 4f
NLRP3
full-length
Δ Exon 5
Δ Exon 5
full-length
kDa
145
50
IB: NLRP3 (cryo-2)
ASC (AL177)
WCL
IP: tagRFP (AB233)
-> NLRP3

Fig. 6a

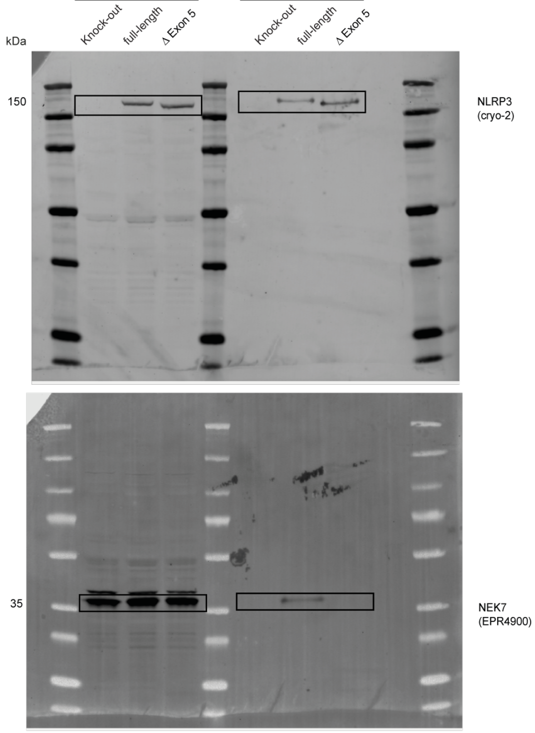

Fig. 6d

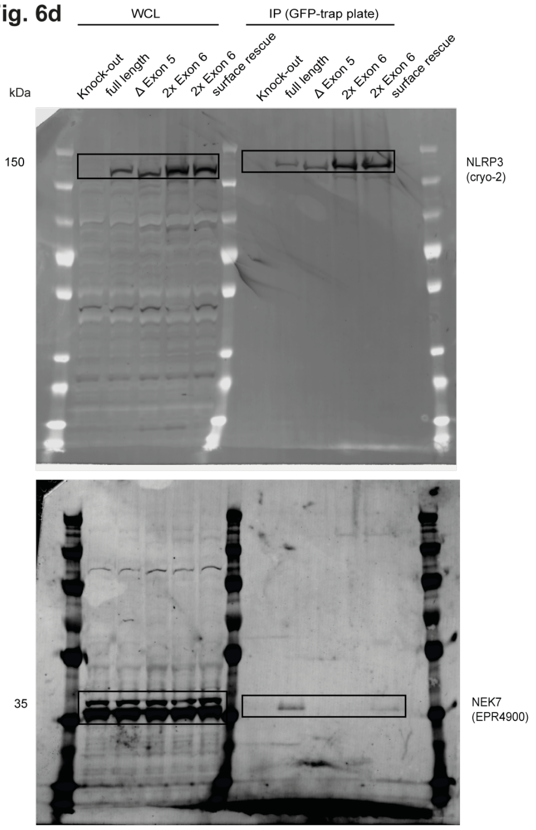

Fig. 6e

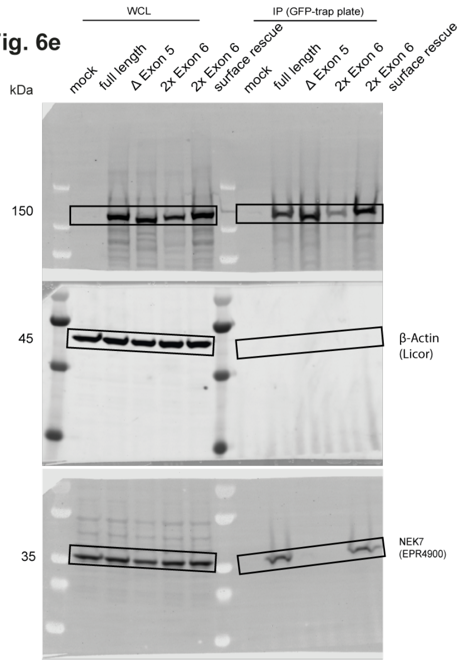

Fig. S6a

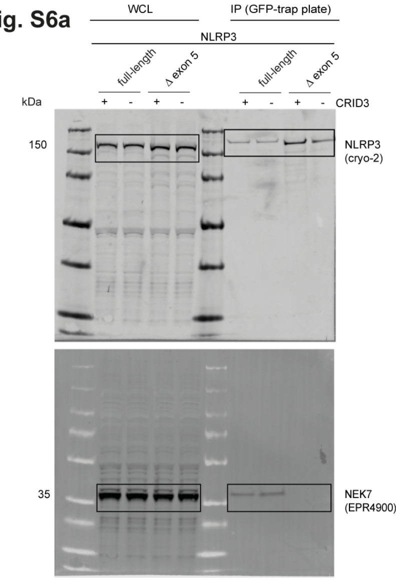

Supplementary Fig. 8
Whole gels and membranes

Tables

	Donor 1	Donor 2	Donor 3	Donor 4	Donor 5
Number of input reads	134943073	16097467	154640058	162862993	176498896
Average input read length	252	252	252	252	252
Uniquely mapped reads	123424533	146198305	144276036	149360709	163061729
Uniquely mapped reads, %	91.46%	90.82%	93.30%	91.71%	91.71%
Average mapped length	245.03	245.74	245.49	245.33	244.5
Mismatch rate per base, %	0.44%	0.42%	0.42%	0.43%	0.43%
Multi-mapping reads	6119358	9613436	4915572	7108225	5922473
Multi-mapping reads, %	4.57%	6.01%	3.21%	4.39%	3.40%
Un-mapped reads, %	3.97%	3.16%	3.50%	3.90%	4.21%

Supplementary Table 1:
Mapping summary of RNA reads

	Full length	Δ Exon 5	2x Exon 6	2x Exon 6 - Exon 5 surface
Sizes (bp)	3108	2937	3108	3108
No. of amino acids	1036	979	1036	1036
Molecular weight (Dalton)	118172.58	111884.42	118181.9	118183.62
No. of − ve charged residue	134	129	135	135
No. of + ve charged residue	122	118	124	122
Theoretical pI	6.22	6.24	6.28	6.17
Instability index	45.6	45.1	43.53	46
Aliphatic index	92.88	92.61	94.86	93.73
GRAVY	-0.203	-0.213	-0.177	-0.198

Supplementary Table 2:
Pysico-chemical properties of NLRP3 isoforms and artificial variants
Physico-chemical characteristics of the NLRP3 full length, Δ exon 5 and the two artificial hybrids containing a doubled exon 6 but no exon 5, or a rescue of all surface amino acids of exon 5 on the doubled exon 6 backbone. Values were calculated with the ExPASy ProtParam tool.

Primer Name	Target	Species	Orientation	Sequence
P1	NLRP3 LRR	Human	Forward	GCTGCAGATCCAGCCCAGCCAG
P2	NLRP3 LRR	Human	Reverse	GTGGTCTTGGCCTGGATGGATCGC
P3	NLRP3 LRR	Mouse	Forward	GCTGCAGTGGCAGCCCAGCCAAC
P4	NLRP3 LRR	Mouse	Reverse	CCCTATACCAGAAGAGCCTCGGCTG
P5	NLRP3 LRR	Pig	Forward	GCTACAGATTGAGCCCAGCCAGC
P6	NLRP3 LRR	Pig	Reverse	ACATTGGCGTCTGACAGCCTTGG
P7	HPRT	Human	Forward	TCAGGCAGTATAATCCAAAGATGGT
P8	HPRT	Human	Reverse	AGTCTGGCTTATATCCAACACTTCG
P9	NLRP3 Exon 4 to 5	Human	Forward	TGCCTGTTCTCATGGattggtg
P10	NLRP3 Exon 5 to 6	Human	Reverse	AGCGCCCCAACcacaatctc
P11	NLRP3 Exon 4 to 6	Human	Forward	CTGTTCTCATGGgttggggc
P12	NLRP3 Exon 6 to 7	Human	Reverse	AGCGCCCCAACcacaatctc
P13	NLRP3 Exon 6 to 8	Human	Reverse	GCCAGAATTCACCAACcagagc
P14	NLRP3 Exon 6	Human	Forward	GAGTGCTGCTTCGACATCTCC
P15	NLRP3	Human	Forward	tttttgccggggcctcttttc
P16	NLRP3	Human	Reverse	aggagatgtcgaagcagcactc
P17	NLRP3 Δexon 5	Human	Forward	aggccgacaccttgata
P18	NLRP3 Δexon 5	Human	Reverse	acagaagtctgattccgaagtcac
P19	HPRT	Human	Forward	atttattttgcatacctaatcatt
P20	HPRT	Human	Reverse	gtaatccagcaggtcagcaaagaa
P21	NLRP3	Human	Forward	gacccagggatgagagtgttgt
P22	NLRP3	Human	Reverse	cccaaccacaatctccgaat
P23	NLRP3 Δexon 5	Human	Forward	caagctcctctcatgctgcc
P24	NLRP3 Δexon 5	Human	Reverse	gagatgtcgaagcagcactcat

Supplementary Table 3:
List of primers used in this study.

Notes

MKMASTRCKLARYLEDLEDVDLKKFKMHLEDYPPQKGCIPLPRGQTEKADHVDLATLMI
DFNGEEKAWAMAVWIFAAINRRDLYEKAKRDEPKWGSDNARVSNPTVICQEDSIEEEW
MGLLEYLSRISICKMKKDYRKKYRKYVRSRFQCIEDRNARLGESVSLNKRYTRLRLIKEH
RSQQEREQELLAIGKTKTCESPVSPIKMELLFDPDDEHSEPVHTVVFQGAAGIGKTILAR
KMMLDWASGTLYQDRFDYLFYIHCREVSLVTQRSLGDLIMSCCPDPNPPIHKIVRKPSRI
LFLMDGFDELQGAFDEHIGPLCTDWQKAERGDILLSSLIRKKLLPEASLLITTRPVALEKL
QHLLDHPRHVEILGFSEAKRKEYFFKYFSDEAQARAAFSLIQENEVLFTMCFIPLVCWIV
CTGLKQQMESGKSLAQTSKTTTAVYVFFLSSLLQPRGGSQEHGLCAHLWGLCSLAADG
IWNQKILFEESDLRNHGLQKADVSAFLRMNLFQKEVDCEKFYSFIHMTFQEFFAAMYYLL
EEEKEGRTNVPGSRLKLPSRDVTVLLENYGKFEKGYLIFVVRFLFGLVNQERTSYLEKKL
SCKISQQIRLELLKWIEVKAKAKKLQIQPSQLELFYCLYEMQEEDFVQRAMDYFPKIEINL
STRMDHMVSSFCIENCHRVESLSLGFLHNMPKEEEEEEEKEGRHLDMVQCVLPSSSHAA
CSHGLVNCHLTSSCCRDIFSVLSSNQSLTELDLSDNSLGDPGIRLLCEGLQHLGCNLRR
LWLGRCGLSHECCFDISLVLSSNQKLVELDLSDNALGDFGIRLLCVGLKHLLCNLKKLWL
VSCCLTSACCQDLASVLSTSHSLTRLYVGENALGDSGVAILCEKAKNPQCNLQKLGLVN
SGLTSVCCSALSSVLSTNQNLTHLYLRGNTLGDKGIKLLCEGLLHPDCKLQVLELDNCNL
TSHCCWDLSTLLTSSQSLRKLSLGNNDLGDLGVMMFCEVLKQQSCLLQNLGLSEMYFN
YETKSALETLQEEKPELTVVFEPSW

Supplementary Note 1:
Protein sequence of NLRP3 2x Exon 6 surface rescue used in this study.
