# Supplementary material for: Alternative splicing regulates stochastic NLRP3 activity
Source: Nat Commun. 2019 Jul 19;10:3238. doi: 10.1038/s41467-019-11076-1 (PMC6642158; doi:10.1038/s41467-019-11076-1)
Supplement: Supplementary file 2 — Reporting Summary [file 41467_2019_11076_MOESM2_ESM.pdf]

nature research

Corresponding author(s): Eicke Latz, Hal M. Hoffman

Reporting Summary

Nature Research wishes to improve the reproducibility of the work that we publish. This form provides structure for consistency and transparency in reporting. For further information on Nature Research policies, see Authors & Referees and the Editorial Policy Checklist.

Statistical parameters

When statistical analyses are reported, confirm that the following items are present in the relevant location (e.g. figure legend, table legend, main text, or Methods section).

n/a	Confirmed	
☐	☒	The exact sample size (n) for each experimental group/condition, given as a discrete number and unit of measurement
☐	☒	An indication of whether measurements were taken from distinct samples or whether the same sample was measured repeatedly
☐	☒	The statistical test(s) used AND whether they are one- or two-sided<br>*Only common tests should be described solely by name; describe more complex techniques in the Methods section.*
☒	☐	A description of all covariates tested
☒	☐	A description of any assumptions or corrections, such as tests of normality and adjustment for multiple comparisons
☐	☒	A full description of the statistics including central tendency (e.g. means) or other basic estimates (e.g. regression coefficient) AND variation (e.g. standard deviation) or associated estimates of uncertainty (e.g. confidence intervals)
☒	☐	For null hypothesis testing, the test statistic (e.g. F, t, r) with confidence intervals, effect sizes, degrees of freedom and P value noted<br>*Give P values as exact values whenever suitable.*
☒	☐	For Bayesian analysis, information on the choice of priors and Markov chain Monte Carlo settings
☒	☐	For hierarchical and complex designs, identification of the appropriate level for tests and full reporting of outcomes
☒	☐	Estimates of effect sizes (e.g. Cohen's d, Pearson's r), indicating how they were calculated
☐	☒	Clearly defined error bars<br>*State explicitly what error bars represent (e.g. SD, SE, CI)*

Our web collection on statistics for biologists may be useful.

Software and code

Policy information about availability of computer code

Data collection	see Methods
Data analysis	see Methods

For manuscripts utilizing custom algorithms or software that are central to the research but not yet described in published literature, software must be made available to editors/reviewers upon request. We strongly encourage code deposition in a community repository (e.g. GitHub). See the Nature Research guidelines for submitting code & software for further information.

Data

Policy information about availability of data

All manuscripts must include a data availability statement. This statement should provide the following information, where applicable:

- Accession codes, unique identifiers, or web links for publicly available datasets
- A list of figures that have associated raw data
- A description of any restrictions on data availability

RNA seq data is deposited at GEO (GSE117206) and has been released.

Field-specific reporting

Please select the best fit for your research. If you are not sure, read the appropriate sections before making your selection.

☒ Life sciences ☐ Behavioural & social sciences ☐ Ecological, evolutionary & environmental sciences

For a reference copy of the document with all sections, see nature.com/authors/policies/ReportingSummary-flat.pdf

Life sciences study design

All studies must disclose on these points even when the disclosure is negative.

Sample size	No sample size calculation was performed.
Data exclusions	Data was not excluded from analysis
Replication	Experiments were reproduced as indicated in the figure legends
Randomization	N/A
Blinding	N/A

Reporting for specific materials, systems and methods

Materials & experimental systems

n/a	Involved in the study
☐	☒ Unique biological materials
☐	☒ Antibodies
☐	☒ Eukaryotic cell lines
☒	☐ Palaeontology
☐	☒ Animals and other organisms
☒	☐ Human research participants

Methods

n/a	Involved in the study
☒	☐ ChIP-seq
☐	☒ Flow cytometry
☒	☐ MRI-based neuroimaging

Unique biological materials

Policy information about availability of materials

Obtaining unique materials	primary human cells were isolated from buffy coats provided by the university hospital blood donation service and pig blood was provided by the central university hospital animal facility and cannot be linked to specific individuals

Antibodies

Antibodies used	see Methods
Validation	All antibodies have been previously used and are noted as suitable for this purpose by the distributor.<br>IL-1beta immunoblots were generated using the detection antibody of the IL-1beta R&D ELISA kit as done before.

Eukaryotic cell lines

Policy information about cell lines

Cell line source(s)	THP1: ATCC<br>murine immortalized macrophages NLRP3 -/-: Latz Lab, UMASS Worcester, USA, produced as Hornung et al. 2008<br>Flp-In 293 T-REx: Life Technologies
Authentication	N/A
Mycoplasma contamination	Not tested

Commonly misidentified lines (See ICLAC register) | N/A

Animals and other organisms

Policy information about studies involving animals; ARRIVE guidelines recommended for reporting animal research

Laboratory animals	BMDMs were generated from WT C57BL/6 mice as indicated in the Methods
Wild animals	N/A
Field-collected samples	N/A

Flow Cytometry

Plots

Confirm that:

- [x] The axis labels state the marker and fluorochrome used (e.g. CD4-FITC).
- [x] The axis scales are clearly visible. Include numbers along axes only for bottom left plot of group (a 'group' is an analysis of identical markers).
- [ ] All plots are contour plots with outliers or pseudocolor plots.
- [ ] A numerical value for number of cells or percentage (with statistics) is provided.

Methodology

Sample preparation	Cells were harvested and analyzed for the expression of the respective NLRP3-fluorescence proteins
Instrument	BD Canto II, BD Aria, MACS-Quant VYB
Software	FlowJo, FACS-Diva (BD)
Cell population abundance	Sorted cell lines were afterwards controlled for expression as indicated
Gating strategy	All cells were included in the analysis and doublets were excluded via FSC-W. Sorting gates were based on cells not expressing the fluorescent protein.

- [x] Tick this box to confirm that a figure exemplifying the gating strategy is provided in the Supplementary Information.
